# L‐arginine metabolism ameliorates age‐related cognitive impairment by Amuc_1100‐mediated gut homeostasis maintaining

**DOI:** 10.1111/acel.14081

**Published:** 2024-01-18

**Authors:** Jiamin He, Tongyao Hou, Qiwen Wang, Qingyi Wang, Yao Jiang, Luyi Chen, Jilei Xu, Yadong Qi, Dingjiacheng Jia, Yanrou Gu, Lidan Gao, Yingcong Yu, Lan Wang, Lijun Kang, Jianmin Si, Liangjing Wang, Shujie Chen

**Affiliations:** ^1^ Department of Gastroenterology Sir Run Run Shaw Hospital, Zhejiang University Hangzhou China; ^2^ Institution of Gastroenterology Zhejiang University Hangzhou China; ^3^ Prevention and Treatment Research Center for Senescent Disease Zhejiang University School of Medicine Hangzhou China; ^4^ Department of Gastroenterology Second Affiliated Hospital of Zhejiang University School of Medicine Hangzhou China; ^5^ Department of General Practice Sir Run Run Shaw Hospital, Zhejiang University School of Medicine Hangzhou China; ^6^ Department of Gastroenterology, Wenzhou People's Hospital Wenzhou Medical University Wenzhou China; ^7^ Third Affiliated Hospital of Shanghai University, Wenzhou Third Clinical Institute Affiliated to Wenzhou Medical University, Wenzhou People's Hospital Wenzhou China; ^8^ NHC and CAMS Key Laboratory of Medical Neurobiology, MOE Frontier Science Center for Brain Research and Brain‐Machine Integration, School of Brain Science and Brain Medicine Zhejiang University Hangzhou China

**Keywords:** aging, Amuc_1100, cognitive impairment, L‐arginine, synaptic function

## Abstract

Aging‐induced cognitive impairment is associated with a loss of metabolic homeostasis and plasticity. An emerging idea is that targeting key metabolites is sufficient to impact the function of other organisms. Therefore, more metabolism‐targeted therapeutic intervention is needed to improve cognitive impairment. We first conducted untargeted metabolomic analyses and 16S rRNA to identify the aging‐associated metabolic adaption and intestinal microbiome change. Untargeted metabolomic analyses of plasma revealed L‐arginine metabolic homeostasis was altered during the aging process. Impaired L‐arginine metabolic homeostasis was associated with low abundance of intestinal *Akkermansia muciniphila* (*AKK*) colonization in mice. Long‐term supplementation of *AKK* outer membranes protein‐Amuc_1100, rescued the L‐arginine level and restored cognitive impairment in aging mice. Mechanically, Amuc_1100 acted directly as a source of L‐arginine and enriched the L‐arginine‐producing bacteria. In aged brain, Amuc_1100 promoted the superoxide dismutase to alleviated oxidation stress, and increased nitric oxide, derivatives of L‐arginine, to improve synaptic plasticity. Meanwhile, L‐arginine repaired lipopolysaccharide‐induced intestinal barrier damage and promoted growth of colon organoid. Our findings indicated that aging‐related cognitive impairment was closely associated with the disorders of L‐arginine metabolism. *AKK*‐derived Amuc_1100, as a potential postbiotic, targeting the L‐arginine metabolism, might provide a promising therapeutic strategy to maintain the intestinal homeostasis and cognitive function in aging.

## Graphical abstract

(1) L‐arginine metabolic pathway mediated the age‐related cognitive impairment during aging process; (2) Amuc_1100 alleviated age‐related cognitive impairment in natural aging mouse and senescence‐accelerated mouse model by L‐arginine/NO pathway.

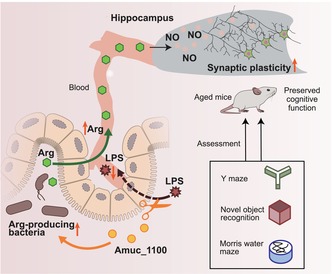

**Abbreviations**
*AKK*
: *Akkermansia muciniphila*
ARCD: age‐related cognitive decline
LPS: lipopolysaccharide
MDA: malondialdehyde
MWM: morris water maze
NO: nitric oxide
NOR: novel object recognition
PCA: principal components analysis
PLS‐DA: partial least squares discrimination analysis
RI: recognition index
Samp8: senescence‐accelerated mouse prone 8
Samr1: senescence‐accelerated resistance 1
SOD: superoxide dismutase

## 1 INTRODUCTION

Due to the increase in our life expectancy, population aging, and healthy aging is a new trend of socialization. While cognitive impairment associated with aging contributes to a common health threat for the elderly population (Bieri et al., 2023).

Cognitive impairment often manifests as neuronal and synaptic alterations. Metabolic stress and the increased oxidative stress are two main contributors to neuronal damage and synaptic plasticity. Nowadays, the concept of “signaling metabolites” is gaining traction. Since blood plasma are easy to obtain, evidence demonstrate the feasibility of using plasma proteomics to identify factors associated with brain aging and cognitive function (Nicholson et al., 2008; Wyss‐Coray, 2016). Villeda et al reported the infusion of plasma from old to young mice accelerated brain aging, while young blood reversed age‐related impairments in cognitive function and synaptic plasticity in mice (Villeda et al., 2011, 2014). The balance of gut microbiota is critical in maintaining plasma metabolic homeostasis and brain health. It was reported that plasma levels of 4‐ethylphenyl sulfate (4EP) were significantly increased in autism spectrum disorder and mice colonized with 4EP‐producing bacteria exhibited reduced myelination of neuronal axons (Needham et al., 2022). And the depletion of acetate‐producing bacteria caused the reduction of synaptophysin (SYP) in the hippocampus, which resulted in learning and memory impairments (Zheng et al., 2021).

*Akkermansia muciniphila* (*AKK*) is a well‐known gut bacterium with broad health‐promoting effects. Several studies had demonstrated an age‐related loss of *AKK* (Biagi et al., 2010; van der Lugt et al., 2019) and proved the protective effect of *AKK* in microbiota‐gut‐brain axis in rodents (Grajeda‐Iglesias et al., 2021; Higarza et al., 2021; van der Lugt et al., 2019; Wu et al., 2020). Live *AKK* would be challenging as being a therapeutic probiotic in clinic due to strict anaerobic growth requirement and potential pathogenicity (Gupta et al., 2021), while postbiotics, acting as non‐viable bacterial cells, bacterial fractions, or cell lysates, could also provide additional physiological benefits to the host (Salminen et al., 2021). Increasing evidence in the literature suggested that postbiotics had important roles in delaying the aging process. Amuc_1100, one of the most abundant proteins of *AKK* outer membranes (van der Lugt et al., 2019), is a natural postbiotic compound that had similar biological activity to *AKK* in modulating host metabolism (Cani & de Vos, 2017; Plovier et al., 2017). Amuc_1100 had an effect on anti‐depression by improving intestinal microbiota, upregulating brain‐derived neurotrophic factor level, and inhibiting neuroinflammatory response (Cheng et al., 2021). Meanwhile, Amuc_1100 had also been reported to attenuate colitis and colitis‐associated colorectal cancer through modulating CD8^+^ T cells (Wang et al., 2020). Amuc_1100 improved gut health and liver steatosis in high‐fat‐fed zebrafish (Zhang et al., 2021). In this sense, to explore the mechanism of Amuc_1100 on the age‐related cognitive impairment holds great promise in translating the results of basic research into applications.

This study aimed to investigate the metabolic alterations of aged mice and identify the effect of Amuc_1100 on the nervous system of aged mice. We found that L‐arginine metabolic pathway mediated the age‐related cognitive impairment during aging process. Amuc_1100 rescued the aging‐induced alteration of the L‐arginine metabolic pathway and protected against high levels of oxidative stress by reducing malondialdehyde (MDA) levels and increasing superoxide dismutase (SOD) enzyme activity in the brain of aged mice, thus improved learning and memory ability, and synaptic function of aged mice. Meanwhile, Amuc_1100‐induced L‐arginine elevation was shown to maintain intestinal stemness and protected the intestinal barrier in vitro. These results will provide a potential therapeutic approach for postbiotics modulation of age‐related cognitive impairment.

## 2 MATERIALS AND METHODS

### 2.1 Animals

In this study, we aimed to establish evidence in age‐related cognitive decline mouse model that *AKK*‐derived Amuc_1100 improved the L‐arginine metabolic dysfunctions by maintaining gut homeostasis to age‐related cognitive impairment.

Young (4‐month‐old) and aged female C57BL/6 mice (10‐month‐old and 20‐month‐old) were obtained from Shanghai Slac Laboratory animal co., LTD. Five‐month‐old male senescence‐accelerated mouse prone 8 (SAMP8) were selected as a cognitive deficits model and senescence‐accelerated resistance 1 (SAMR1) mice of the same age were selected as the negative control. These animals were obtained from Beijing HFK Bio‐Technology.co., LTD. For consistency, all mice used in this study were kept under standard conditions. The mice were divided into different groups balanced by weight: young (4‐month‐old) group and aged (20‐month‐old) group; SAMR1 group and SAMP8 group; aged + PBS‐treated (200 μL), aged + Amuc_1100‐treated (3 μg/200 μL); SAMP8 + PBS‐treated (200 μL), SAMP8 + Amuc_1100‐treated (3 μg/200 μL). For the intervention queue, all mice were orally gavaged every other day. The animals were sacrificed after 6 months of administration. The intestine and brain of the animals were isolated on ice for further experiments. Plasma and fecal were selected. Sample sizes were maintained at 5 to 9 mice per group, according to previous similar studies. Each mouse participated in behavioral experiments, which N for each group was shown in the figure legend. For each group of mice, the order of testing was randomized. All animal studies were approved by the “Animal Care and Use Guidelines” of Zhejiang Chinese Medical University Laboratory Animal Research Center (approval numbers: 20210705‐22).

### 2.2 Novel object recognition

Firstly, each mouse freely explored the open‐field arena for 1 h without objects to achieve the habituation of the experimental environment. Then, each mouse was presented with two identical objects for 5 min. Each mouse rested for 30 min and then they returned to be re‐exposed to the old object and a completely new object. Video recording equipment was required during all phases. The recognition index (RI) is calculated as: RI = new object/(new object + old object) × 100%.

### 2.3 Y maze (electrical stimulation)

The Y maze consisted of three identical arms with light bulbs at the end that could be varied in light and darkness. And the bottom of the Y‐maze was lined with copper rods that could be electrically stimulated. The bright arm was set to a safe zone without electrical stimulation, while the dark arm was set to a stimulation zone with electrical stimulation. Mice preferred to stay in the dark arm, but the lighted arm was the safe zone. Mice were trained by varying the lighting of the safe and stimulation zones. The mice were considered to have responded correctly if it escaped from the dark zone to a safe zone within 10 s. In 10 consecutive foot stimuli, the mouse was considered to have learned if they achieved 9 or more correct responses. To assess the learning capacity, the total number of stimuli to reach the criterion during training were recorded. After 24 h of training, the memory for the Y‐maze was tested. During the test procedure, the mice received a total of 30‐feet stimuli and the number of errors in the 30 stimuli was recorded.

### 2.4 Morris water maze (MWM) testing

Morris water maze testing was performed as previously described to assess spatial learning (Velazquez et al., 2019). The mouse was tested in a circular tank with extramaze cues. During the whole test, the water surface was kept at 25°C and the platform was submerged at 1.5 cm below the water surface. For training period, each mouse would be given 1 min to find the location of the platform, and if it failed, it was guided to the location of the platform and maintained for 10 s on the platform. And each mouse received three trials per day for six consecutive days of training at the same time. A probe trial was conducted 24 h after the last training trial. In the probe test, the mouse was allowed to swim freely for 60 s without the platform. All tests were fully videotaped, and the data was recorded via the SMART Video Tracking System for further analysis.

### 2.5 Y‐maze forced alternation

The Y‐maze forced alternation relies on the tendency of mice to explore new environments to assess spatial recognition memory in rodents. Firstly, one arm was blocked as the novel arm. The mouse was placed at the end of the start arm facing away from the center and allowed to explore the two open arms for 5 min. After rest, the mouse was placed at the end of the start arm and allowed to explore all three arms of the Y‐maze for 5 min during the test. All tests were fully videotaped, and the data were recorded via the SMART Video Tracking System for further analysis.

### 2.6 Golgi–cox staining

Golgi‐cox staining was performed with the assistance of the Servicebio (Wuhan, China). An area of brain tissue was selected for 1000× imaging using an Eclipse Ci‐L photomicrograph microscope. The number of dendritic spines within 30–90 μm length of the 2nd or 3rd dendritic branch on the intact neuron was measured separately. A total of 10 neurons per parameter for each animal were analyzed, and three animals were used for each group.

### 2.7 Lipopolysaccharide (LPS) measurement

The concentration of circulating plasma LPS was measured using mouse LPS ELISA Kit (Cat. ml037221‐1, Mlbio, China). Then it was measured at 450 nm using a spectrophotometer.

### 2.8 Plasma metabolomics

Blood was collected after the mice were euthanized and was centrifuged at 3500 *g* at 4°C for 15 min. 50 μL plasma sample was added with 200 μL solution (acetonitrile: methanol = 1:1(v:v)) containing 0.02 mg/mL internal standard (L‐2‐chlorophenylalanine) to extract metabolites in a 1.5 mL centrifuge tube. Samples were vortexed for 30 s and then sonicated for 30 min (4°C, 40 KHz). The samples were left at −20°C for 30 min to allow the proteins to settle. Then the samples were centrifuged for 15 min (4°C, 13,000 *g*). The supernatant was blown dry under nitrogen. The sample was then reconstituted with 60 μL of solution (acetonitrile: water = 1:1) and extracted by cryo‐sonication for 5 min (5°C, 40 KHz), followed by centrifugation at 13,000 *g* and 4°C for 10 min. The supernatant was transferred to sample vials for LC–MS/MS analysis, which was conducted at Shanghai MajorBio (China).

### 2.9 Detection the L‐Arginine by LC–MS/MS

Plasma samples were prepared as described above. Precisely weighed 15 mg of fecal sample or hippocampal tissue. The samples were ground for 6 min (−10°C, 50 Hz) with 150 μL of extraction solution in a freeze grinder and sonicated for 30 min (4°C, 40 KHz). Then the samples were centrifuged at 4°C for 5 min at 13,000 rcf, and the supernatant was diluted with 100 μL of acetonitrile, vortexed, and mixed. The supernatant was centrifuged at 13,000 rcf for 5 min at 4°C, and the supernatant was diluted 5 times. Then the supernatant was transferred to sample vials for LC–MS/MS analysis according to the described previously (Mao et al., 2022), which was conducted at Zhejiang Academy of Agricultural Sciences (Hangzhou, China).

### 2.10 In vitro digestion model

The simulated gastric fluid (Cat. R22155) and simulated intestinal fluid (Cat. R22156) were purchased from Shanghai yuanye Bio‐Technology Co., Ltd. Amuc_1100 or PBS were then digested by simulated gastric fluid and simulated intestinal fluid according to the previous literature (Liu et al., 2019). Simply, add 30 μL of Amuc_1100 or PBS to the 1 mL of simulated gastric fluid or simulated intestinal fluid, respectively. Each group was digested separately in a water bath at 37°C. After 3 h, 1 mL of the mixture was removed and inactivated in a boiling water bath for 5 min. The mixture was then centrifuged at 4500 r/min for 10 min. Then Amuc_1100 gastric extraction sample, PBS gastric extraction sample, Amuc_1100 intestinal extraction sample, and PBS intestinal extraction sample were performed separately for L‐arginine detection by LC–MS/MS.

### 2.11 Detection of nitric oxide (NO)

The hippocampal tissue was homogenized in PBS buffer according to 1:10, and then was centrifuged at 10,000 *g* for 10 min. The supernatant was taken for measurement according to the Nitric Oxide (NO) Colorimetric Assay Kit (Nitrate Reductase Method) (Cat. A012‐1, Nanjing Institute of Bioengineering, China), and part of the supernatant was retained for protein concentration determination (Cat. PC0020, Solarbio, China). The NO content in the hippocampal tissue is shown by μmol/gprot.

### 2.12 Assessment on markers of oxidative stress

Superoxide dismutase (SOD) (Cat. A001‐1) and malondialdehyde (MDA) (Cat. A003‐1) kits were purchased from Nanjing Jiancheng Bioengineering Institute and conducted according to the manufacturer's guidelines.

### 2.13 Colon organoid culture

Cut mouse colon samples into 4–5 mm and then were incubated in 20 mM EDTA in PBS for 30 min at 4°C. For mouse organoids, 200 crypts per well in 24‐well plates were suspended in 50 μL Matrigel composed of 25% IntestiCult OGM Mouse Kit (Cat. 06005, Stemcell) and 75% growth factor‐reduced Matrigel (Cat. 354230, Corning). The Matrigel suspension was placed at 37°C for 30 min to fix. Then 500 μL fresh complete medium with 10 mM Y‐27632 (Cat. S1049, Sellceck, China) was added to the Matrigel for the first 48–72 h of culture. Day 3, colonoids were treated with L‐arginine (0.4 mM; Cat. PHR1106, Sigma, USA) or PBS. The complete medium was replaced every 2 days. Images were taken on Day 3, Day 5, and Day 7. We captured the images of colon organoids in each well using microscope (Carl Zeiss). Then the diameter and buds of colon organoids was analyzed using the Zen image program (Carl Zeiss).

### 2.14 Culture of primary neurons

E14‐16 embryos were used to obtain embryonic hippocampal primary neuron. After the removement of the meninges, and hippocampal bodies were dissected into small pieces with 0.025% trypsin at 37°C for 15 min. The cell suspension was filtered through a 70 μm cell filter, then the cells were inoculated at 20,000 cells per well on PDL‐coated coverslips in 12‐well plates using F‐12K medium with 10% FBS; After 6 h, they were replaced with B‐27 and L‐glutamine (2 mM; Cat. 35050061, GlutaMAX, Invitrogen) containing Neurobasal medium. Primary neurons were cultured until day 14 when Sodium Nitroprusside (SNP, nitric oxide donor) (20 μM; Cat. S0015, Beyotime, Shanghai) or an equal volume of PBS was added, respectively, and subsequent immunofluorescence staining was performed after 24 h.

### 2.15 Immunohistochemistry

4% paraformaldehyde was used to fix the colon tissues. Then colon sections were embedded in paraffin and stained with hematoxylin and eosin. Inflammation scoring was conducted by pathologists.

### 2.16 Immunofluorescence

For tissue immunofluorescence, the brain slices were dewaxed to water for antigen repair, then blocked with 5% bovine serum albumin (BSA) for 30 min at room temperature, followed by incubation with primary antibodies at 4°C overnight.

For cellular immunofluorescence staining, cells cultured on plastic coverslips were fixed with 4% paraformaldehyde for 10 min and incubated in 5% BSA and 0.2% Triton‐X 100 in PBS for 30 min, followed by incubation with primary antibodies at 4°C overnight.

The primary antibody anti‐NeuN (1:200, Cat. GB11138, Servicebio, China), anti‐Iba1 (1:200, Cat. GB11105, Servicebio, China), anti‐GFAP (1:200, Cat. GB11096, Servicebio, China), anti‐Nos3 (1:400, Cat. GB12086, Servicebio, China), anti‐CD31 (1:400, Cat. GB12063, Servicebio, China), anti‐Synaptophysin (SYN‐1) (1:400, Cat. GB11553, Servicebio, China), anti‐ZO‐1 (1:200, Cat. GB111402, Servicebio, China) antibody, and anti‐map2 (1:400, Cat. GB11128‐2, Servicebio, China) were used. Then the sections were incubated with the secondary antibodies at 37°C for 1 h. The secondary antibody Alexa Fluor® 594 (1:400, Cat. 111‐585‐003, Jackson) and Alexa Fluor® 488 (1:400, Cat. GB25303, Servicebio, China) were used. Finally, DAPI was used to stain cell nuclei for the sections (Cat. FD8396, Fude Biological technology, China). Photographs were taken using a Leica TCS SP8‐DIVE two‐photon microscope.

### 2.17 Monolayer intestinal epithelial cell model

Caco‐2 cell (Cat. SCSP‐5027, Shanghai, China) was obtained from national collection of authenticated cell cultures and used within 10 passages. 1× 10^4^ cells/well were seeded on the 24‐well chambered slides. Then they were treated with LPS (10 μg/mL, Cat. S1732, Beyotime, Shanghai, China) or LPS + PBS or LPS + L‐arginine (0.4 mM, Cat. A118651, aladin, Shanghai, China) for 24 h.

### 2.18 16S rRNA sequencing

SPINeasy DNA Kit for Feces kits (Cat. 116531060, MP Biomedicals, Beijing, China) was used to extract bacterial DNA. The Illumina MiSeq platform (Illumina, San Diego, USA) was used to perform the DNA sequence according to the standard protocols of Shanghai MajorBio (China). The data were analyzed through the service majorbio platform (cloud.majorbio.com) as before (Qi et al., 2023). Briefly, OTU clustering of non‐repetitive sequences (excluding single sequences) was performed according to 97% similarity by Uparse (version 7.0.1090 http://drive5.com/uparse/), and chimeras were removed. Taxonomic annotation of OTUs using the RDP classifier ratio against the Silva 16S rRNA gene database with a 70% confidence threshold. The similarity of microbial community structure between samples was examined using PCA based on the ANOSIM algorithm.

### 2.19 Quantitative real‐time PCR

RNA was extracted from the tissues of mice by using TRIzol reagent (Cat. AG21101, AG, Hunan, China) and reverse transcribed using the PrimeScript™ RT reagent Kit (Cat. RK20429, ABclonal, Wuhan, China). RT‐qPCR was performed using qPCR SYBR Green Master Mix (Cat. RK21203, ABclonal, China) DNA in a ROCHE LightCycler®480II System (Rotor Gene 6000 Software, Australia) in triplicate. The relative abundance was calculated using the −ΔCt method or fold change. The specific primers used in this study are listed in Table S1. β‐actin serves as a control gene.

### 2.20 Statistical analysis

Data were presented as means ± SD. GraphPad Prism 9.0 software (GraphPad Software, Inc., La Jolla, CA, USA) was used to perform the statistical analyses. Before statistical analysis, data were analyzed for normality using the Shapiro–Wilk test or the Kolmogorov–Smirnov test. For comparisons of two different groups, Student's *t* test or nonparametric test was used. For comparisons among multiple groups, analysis of variance (ANOVA), or the Kruskal–Wallis test was used. *p*‐value <0.05 was considered statistically significant.

## 3 RESULTS

### 3.1 Aging impaired the learning and memory ability

**FIGURE 1:**
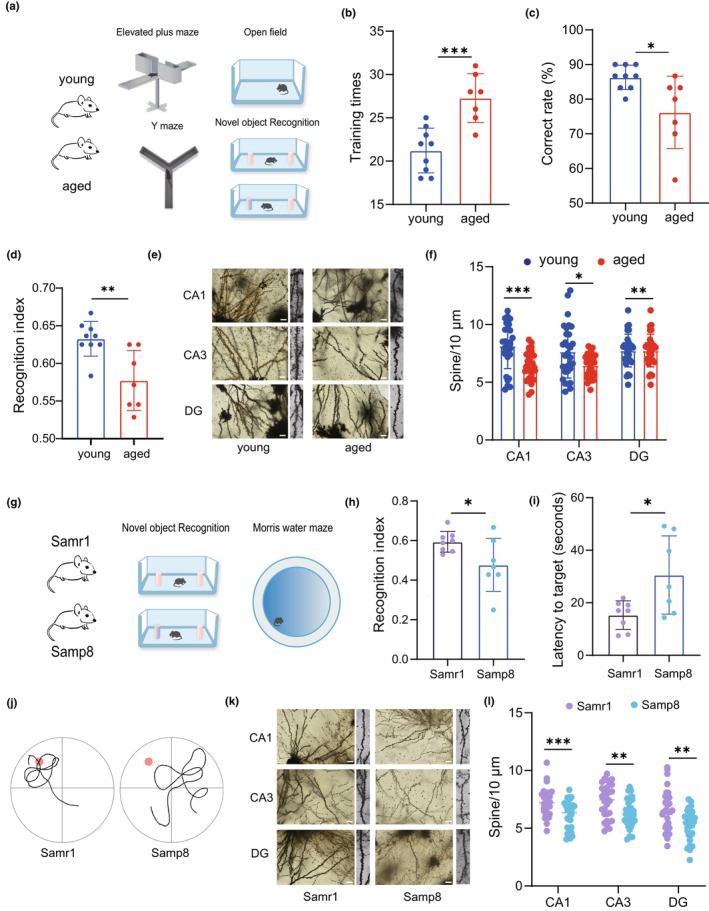
Aging impaired the learning and memory ability. (a) Schematic diagram showing the strategy in young (4‐month‐old) and aged (20‐month‐old) mice. (b) Training times of the mice during the electrical stimulation Y maze (*p =* 0.0005) (*n* = 9 mice for the young group; *n* = 7 mice for the aged group). (c) Correct rate of the mice in the electrical stimulation Y maze (*p =* 0.0161) (*n* = 9 mice for the young group; *n* = 7 mice for the aged group). (d) NOR test recognition index of mice (*p =* 0.0035) (*n* = 9 mice for the young group; *n* = 7 mice for the aged group). (e) Representative Golgi–Cox‐staining images showing the density and morphology of dendritic spines in the cone cell layer of the murine hippocampal CA1, CA3 and DG region in each group. Scale bar, 10 μm. (f) Analysis of total dendritic spine density of the CA1, CA3 and DG region in each group (*n* = 3 mice for each group, total 180 neurons). (g) Schematic diagram showing the strategy in Samr1 (10‐month‐old) and Samp8 (10‐month‐old) mice. (h) NOR test recognition index of mice (*n* = 8 mice for the Samr1 group; *n* = 7 mice for the Samp8 group;) (i) Latency of first time to enter the target (*p =* 0.0177) in the probe trial of the MWM test (*n* = 8 mice for the Samr1 group; *n* = 7 mice for the Samp8 group). (j) The target entries (*p =* 0.0413) in the probe trial of the MWM test (*n* = 8 mice for the Samr1 group; *n* = 7 mice for the Samp8 group). (k) Representative Golgi‐Cox‐staining images showing the density and morphology of dendritic spines in the cone cell layer of the murine hippocampal CA1, CA3, and DG region in each group. Scale bar, 10 μm. (l) Analysis of total dendritic spine density of the CA1, CA3, and DG region hippocampal region in each group (*n* = 3 mice for each group, total 180 neurons). Data were represented as mean ± SD. Comparisons were performed by unpaired two‐tailed *t* test or nonparametric Mann–Whitney test (b–d, f, h–j and l). *p < 0.05, **p < 0.01, and ***p < 0.001; ns, not significant.

Firstly, we examined age‐related behavioral performance in young and aged mice (Figure 1a). In Y‐maze (electrical stimulation) test, the learning ability of the aged mice was impaired. Aged mice needed more training times to achieve learning criterion during the training sessions (Figure 1b), along with decreased accurate rate in the trial (Figure 1c). Additionally, aged mice spent remarkably less time and entries in new object during novel object recognition (NOR) test (Figure 1d). We also tested the elevated plus maze and open filed, while there were no significant differences between young and aged mice (Figure S1a,b). Due to the importance of hippocampal synaptic plasticity for the learning and memory (Parra‐Damas et al., 2017), we subsequently focused on alterations in synaptic plasticity. Golgi staining showed that the density of dendritic spines in aged mice decreased, compared with young mice (Figure 1e,f).

Samp8 is a model of memory deficits associated with aging, we next examined age‐related behavioral performance in this specific model on 10‐month‐old (Figure 1g). As we expected, the 10‐month‐old Samp8 mice showed impaired the learning and memory function compared with Samr1 mice, assessed by NOR (Figure 1h) and MWM test (Figure 1i,j). And the aged Samp8 mice showed reduced density of dendritic spines compared with the Samr1 mice at the same age by Golgi staining (Figure 1k,l).

These findings indicated that aged mice exhibited typical poor hippocampus‐dependent learning and memory ability instead of emotional disorders.

### 3.2 Aging altered the metabolic profile and gut microbial composition

**FIGURE 2:**
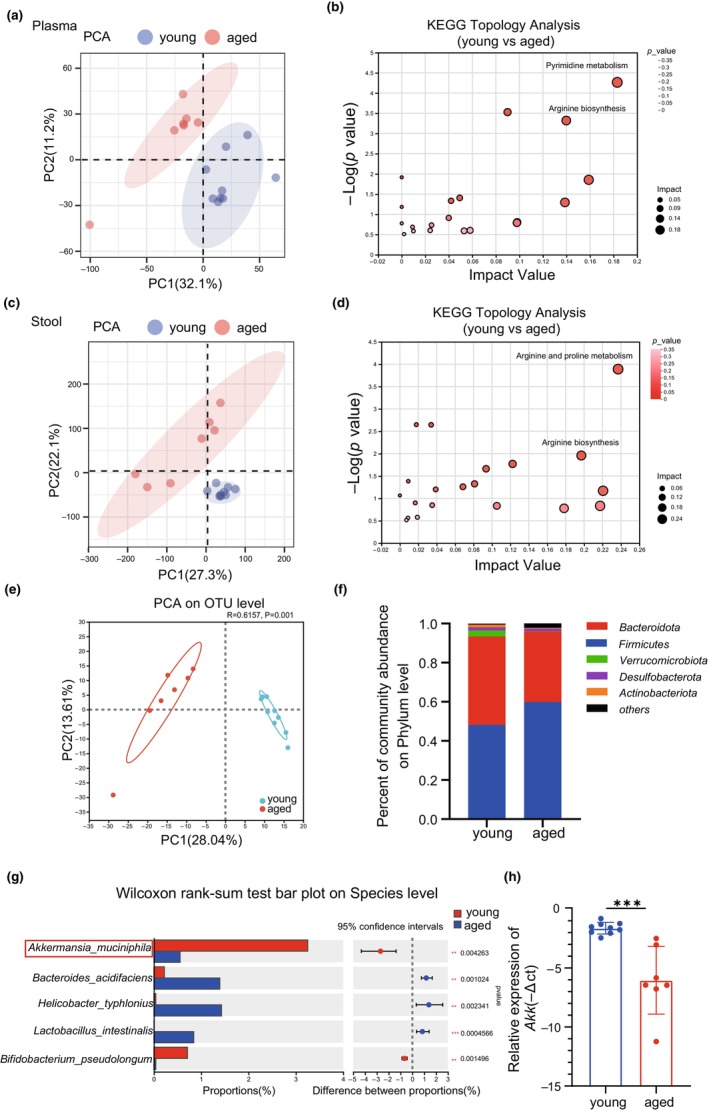
Aging altered the metabolic profile and gut microbial composition. (a) The principal components analysis (PCA) of the effects of aging on plasma metabolome in young and aged mice. (b) KEGG enrichment analysis of plasma metabolome in young and aged mice. (c) PCA of the effects of aging on intestinal metabolome in young and aged mice. (d) KEGG enrichment analysis of stool metabolome in young and aged mice. (e) PCA showed variations of gut microbiota composition between two groups, and each character represented a sample. (f) The percent of different taxa at the Phylum level between two groups. (g) Significant bacterial differences at Species level between two groups. (h) The abundance of *AKK* in stool of two groups. Comparisons were performed by unpaired two‐tailed *t* test (h). *p < 0.05, **p < 0.01, and ***p < 0.001; ns, not significant.

To investigate the metabolic adaptations during aging, we analyzed plasma metabolome using LC–MS/MS. We found aging‐induced significant differences in plasma metabolites, compared with young mice (Figure 2a). And several pathways exhibited significant alterations in the comparison of young versus aged mice, such as pyrimidine metabolism and L‐arginine biosynthesis (Figure 2b). Since the metabolism of L‐arginine played a key role in the progression from healthy to mild cognitive impairment and to Alzheimer's disease in naturally aging populations (Xie et al., 2021), we went further to explore the reasons for the altered L‐arginine metabolic pathway due to aging. Most of circulating metabolites are produced by microbiota and hosts in the digestive tract (Zhang et al., 2023). Then, we performed intestine metabolome using LC–MS/MS. The principal components analysis (PCA) showed aging‐induced significant differences in intestine metabolites (Figure 2c) and L‐arginine metabolic pathway also underwent significant changes in the gut during the aging process (Figure 2d), suggesting that changes in the gut metabolome may play an important role in the plasma metabolome. The gut metabolome is inherently linked to the gut microbiome (Li et al., 2022). As we know, the imbalance of gut microbiota occurs during the aging process (Ling et al., 2022). Then, we explored the composition of the gut microbiota between young and aged mice groups by fecal 16S rRNA sequencing. PCA highlighted that aging reshaped the intestinal microbiota homeostasis (Figure 2e). We found the composition of gut microbiota changed at phylum level between young and aged mice (Figure 2f). Compared to young mice, aged mice harbored a distinctively lower species level of *AKK* (Figure 2g). And qPCR confirmed that *AKK* abundance was decreased in aged mice (Figure 2h). These results suggested that aging altered the overall structure and composition of gut microbiota, particularly resulting in the low abundance of *AKK*, which might play a vital role in L‐arginine‐related pathway.

### 3.3 Amuc_1100 rescued L‐arginine metabolic disorders to improve the learning and memory ability of aged mice

**FIGURE 3:**
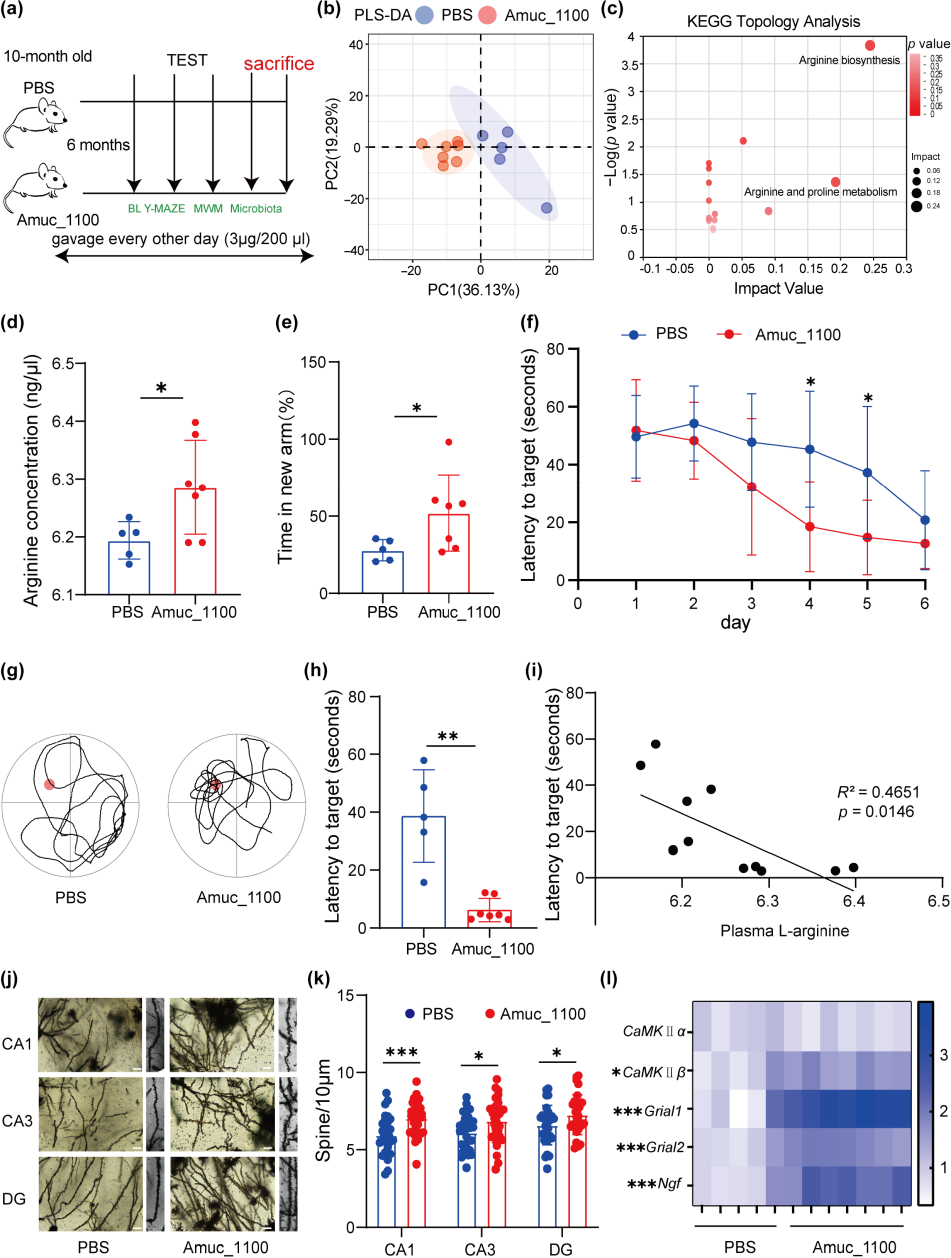
Amuc_1100 rescued arginine metabolic disorders to improve the learning and memory ability of aged mice. (a) Schematic diagram showing the strategy in PBS and Amuc_1100 treated aged mice. (b) PLS‐DA showing the effects of Amuc_1100 on plasma metabolome in PBS and Amuc_1100 treated aged mice. (c) KEGG enrichment analysis of plasma metabolome. (d) The plasma level of L‐arginine in PBS and Amuc_1100 treated aged mice (*p =* 0.0388) (*n* = 5 mice for the PBS group; *n* = 7 mice for the Amuc_1100 group). (e) In the Y‐maze forced alternation test, the time spent in the new arms (*p =* 0.043) of two groups (*n* = 5 mice for the PBS group; *n* = 7 mice for the Amuc_1100 group). (f) Latency of first time to enter the target during the training period in a MWM test (*n* = 5 mice for the PBS group; *n* = 7 mice for the Amuc_1100 group). (g) Representative trajectory diagram in the probe trial of the MWM test. (h) Latency of first time to enter the target (*p =* 0.0025) in the probe trial of the MWM test (*n* = 5 mice for the PBS group; *n* = 7 mice for the Amuc_1100 group). (i) Correlation analysis of the plasma level of L‐arginine and the latency of first time to enter the target (*n* = 11 Spearman *r* ^2^ = 0.4651, *p* = 0.0146). (j) Representative Golgi‐Cox‐staining images showing the density and morphology of dendritic spines in the cone cell layer of the murine hippocampal CA1, CA3, and DG region in each group. Scale bar, 10 μm. (k) Analysis of total dendritic spine density of the CA1, CA3, and DG region hippocampal region in each group (*n* = 3 mice for each group, total 180 neurons). (l) Relative mRNA levels of *Grial1*, *Grial2*, *Ngf*, *Bdnf*, *CaMKIIα*, and *CaMKIIβ* gene in hippocampal tissues by RT‐PCR (*n* = 5 mice for the PBS group; *n* = 7 mice for the Amuc_1100 group). Actin was regarded as internal reference. Data were represented as mean ± SD. Comparisons were performed by unpaired two‐tailed *t* test or nonparametric Mann–Whitney test (d, e, h, k and l); two‐way analysis of variance with Tukey's multiple comparisons test (f); Spearman correlation analysis (i). *p < 0.05, **p < 0.01, and ***p < 0.001; ns, not significant.

Amuc_1100, one of the outer membrane proteins of *AKK*, is natural postbiotic compound (van der Lugt et al., 2019). We then sought to determine whether Amuc_1100 could rescue the L‐arginine metabolic alterations caused by aging and improve the learning and memory ability in aged mice. We first constructed Amuc_1100 protein according to previous literature reports (Wang et al., 2020) (Figure S2), then treated aged mice with Amuc_1100 or PBS for 6 months (Figure 3a). We investigated the metabolic adaptations after the treatment of Amuc_1100, and we found Amuc_1100 changed significant differences in plasma metabolites, compared with aged mice (Figure 3b). Based on KEGG database, we found that L‐arginine biosynthesis and metabolism pathway was enriched in Amuc_1100 group (Figure 3c), especially higher level of L‐arginine in plasma (Figure 3d). Then cognitive‐related behavior tests were conducted after the interval. Very interestingly, *AKK*‐derived Amuc_1100 treated aged mice spent remarkably more time and entries in new arm during Y‐maze forced alternation test, compared with PBS‐treated aged mice (Figures 3e and S3a). In MWM test, a better learning performance was showed in the Amuc_1100 treated aged mice during the training sessions (Figure 3f), along with more crossing times and a shorter latency in finding the target platform in the probe trial (Figure 3g,h). And we observed that the learning and memory ability positively correlated with plasma level of L‐arginine in aged mice (Figure 3i). The basal locomotive behavior between the aged mice treated with Amuc_1100 or PBS showed no significant differences (Figure S3b), indicating that the improvement of the learning and memory ability by Amuc_1100 did not result from altered motor ability.

NeuN staining showed no visible neuronal variety in the hippocampus of aged mice treated with Amuc_1100 or PBS (Figure S3c), and the morphology of microglia did not change obviously in our study (Figure S3d,e). Golgi staining revealed that Amuc_1100 increased the density of dendritic spines (Figure 3j,k). Moreover, the markers reflected synaptic plasticity were also improved after Amuc_1100 treatment (Figure 3l).

We further validated the role of Amuc_1100 on the Samp8 senescence model mouse (Figure S4a). As we expected, the level of plasma L‐arginine was higher in Amuc_1100 treated aged Samp8 mice (Figure S4b) and the application of Amuc_1100 also improved the learning and memory function in aged Samp8 mice, assessed by NOR (Figure S4c) and MWM test (Figure S4d–f). To determine the function of Amuc_1100 on hippocampal synaptic plasticity in aged Samp8 mice, Golgi stainings were conducted. The results showed that Amuc_1100 increased the density of dendritic spines in aged Samp8 mice (Figure S4g,h). And those markers reflected synaptic plasticity were also increased upon Amuc_1100 treated (Figure S4i).

Above data suggested that the *AKK*‐derived Amuc_1100 improved learning and memory ability and synaptic plasticity in aged mice probably due to the improvement of L‐arginine metabolism.

### 3.4 NO improved impaired synaptic function

**FIGURE 4:**
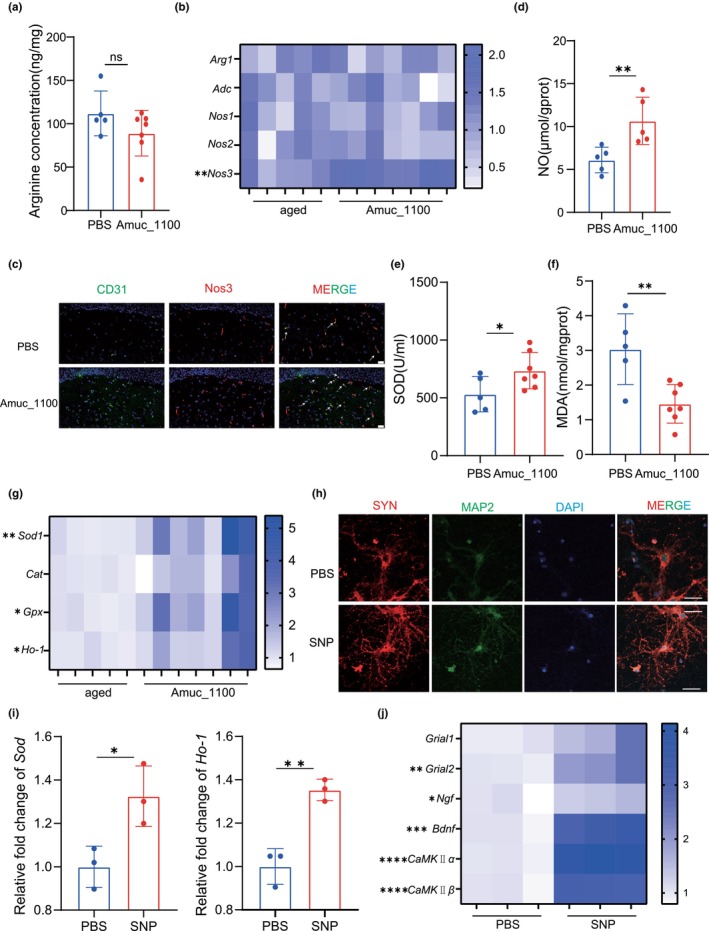
NO improved impaired synaptic function. (a) The level of L‐arginine in hippocampus of two groups (*n* = 5 mice for the PBS group; *n* = 7 mice for the Amuc_1100 group). (b) Relative mRNA levels of *Arg1*, *Adc*, *Nos1*, *Nos2*, *and Nos3* gene in the hippocampal tissues. (c) Representative immunofluorescence image of Nos3/CD31 in hippocampus of two groups. Scale bar, 20 μm. (d) The level of NO in hippocampus of two groups. (e, f) The SOD activity and MDA levels in the hippocampal tissues of two groups. (g) Relative mRNA levels of *Sod1*, *Cat*, *Gpx*, and *Ho‐1* gene in the hippocampal tissues of two groups. (h) Representative immunofluorescence image of SYP‐1/Map in primary mature neurons. Scale bar, 20 μm. (i) Relative mRNA levels of *Sod1* and *Ho‐1* gene in primary mature neurons. (j) Relative mRNA levels of *Grial1*, *Grial2*, *Ngf*, *Bdnf*, *CaMKIIα*, and *CaMKIIβ* gene in primary mature neurons by RT‐PCR. Data were represented as mean ± SD. Comparisons were performed by unpaired two‐tailed *t* test or nonparametric Mann–Whitney test (a–c, e–g, i, and j). *p < 0.05 and **p < 0.01; ns, not significant.

We found aging resulted in the accumulation of L‐arginine in hippocampus (Figure S5a). L‐arginine is a semi‐essential amino acid that can be metabolized by nitric oxide synthase (NOS) to produce nitric oxide (NO) and L‐citrulline, by arginase to form L‐ornithine and urea, and by arginine decarboxylase to generate agmatine and carbon dioxide (Wu & Morris, 1998). NOS has an approximately 1000‐fold greater affinity for L‐arginine than arginase, and the low endogenous agmatine levels in the mammalian brain (Li et al., 1994), suggesting the predominance of the NOS pathway in arginine metabolism under physiological condition. We found the activity of NOS pathway was impaired during senescence (Figure S5b–f). The treatment of Amuc_1100 decreased the level of L‐arginine in the hippocampus (Figure 4a). Then we measured the expression of L‐arginine metabolism‐related enzymes in hippocampus tissue. We found Amuc_1100 could improve the expression of Nos3 (eNOS) (Figure 4b,c). Considering that elevated citrulline was also observed in plasma, we hypothesized that the L‐arginine‐to‐nitric oxide pathway was activated in hippocampus. NO was reported to play a vital role in synaptic plasticity and learning and memory (Paul & Ekambaram, 2011). The content of NO in hippocampus was also confirmed, and Amuc_1100 treated aged mice showed higher level of NO in hippocampus (Figure 4d). SOD, CAT, GSH‐PX, and MDA are important indicators for assessing brain antioxidant capacity. Increasing oxidative stress was observed in the brain of aged mice (Figure S6a–c) and the application of Amuc_1100 could improve the antioxidant capacity and reduce oxidative stress levels (Figure 4e,f), which played an important regulatory role in eNOS activity. And qPCR confirmed that Amuc_1100 increased the mRNA levels of *Sod1*, *Gpx*, and *Ho‐1* in the hippocampus tissue of aged mice (Figure 4g). In vitro, we selected 14‐day primary mature neurons as the study subjects. Compared with the control, primary neurons co‐cultured with sodium nitroprusside (SNP, nitric oxide donor) had more SYN‐1 positive spots and exhibited improved synaptic damage (Figure 4h). SNP treated primary neurons showed higher antioxidant capacity (Figure 4i), and the markers reflected synaptic plasticity were also improved after SNP treatment (Figure 4j). These results supported the hypothesis that Amuc_1100 improved the antioxidant capacity of the brain and that NO, derived from L‐arginine by eNOS, protected against cognitive impairment and synaptic damage.

### 3.5 Amuc_1100 maintained intestinal homeostasis in aged mice

**FIGURE 5:**
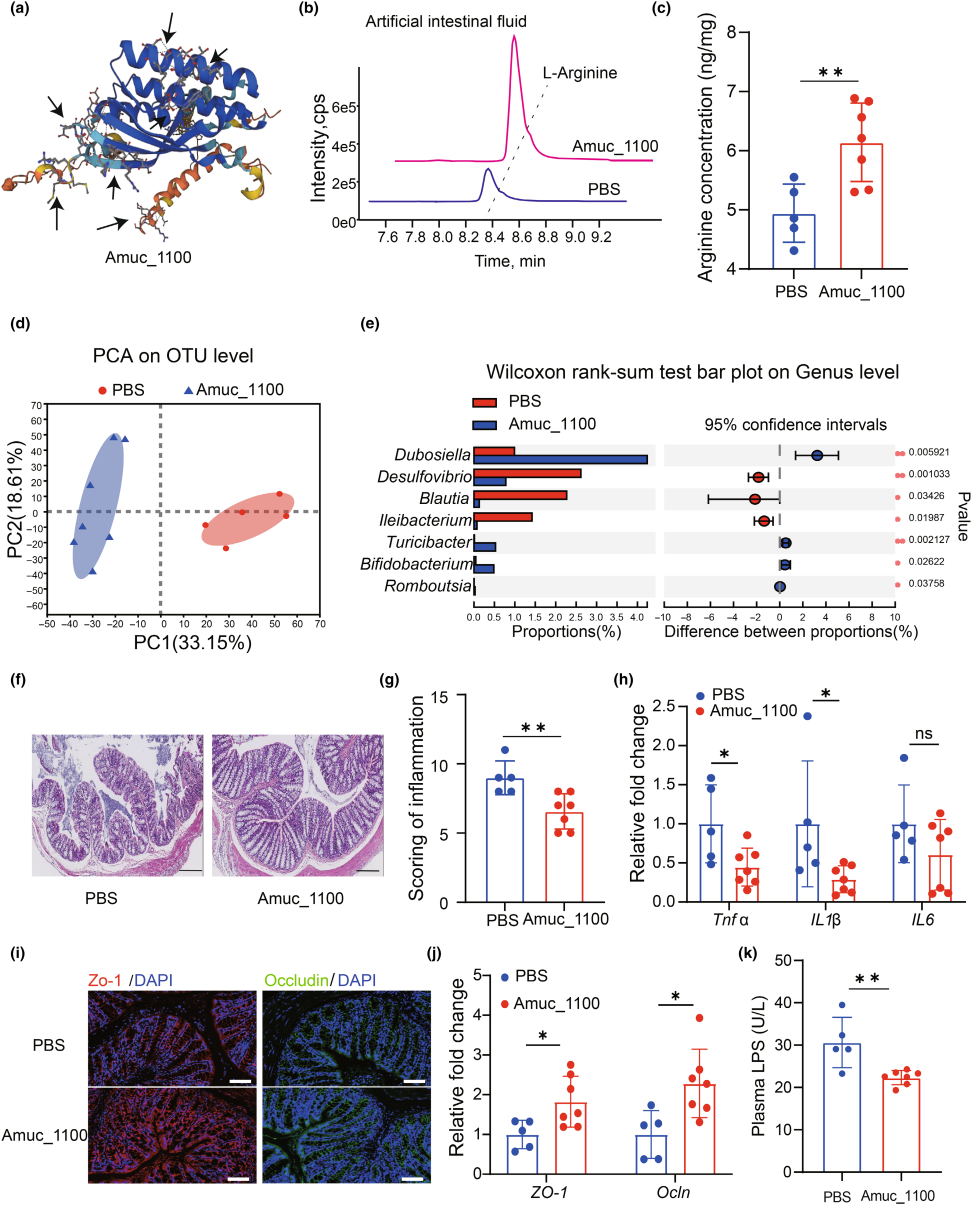
Amuc_1100 maintained intestinal homeostasis in aged mice. (a) The structural composition of Amuc_1100. L‐arginine is indicated by the arrow. (b) LC–MS/MS identification of L‐arginine between PBS and Amuc_1100 group in artificial intestinal fluid in vitro. (c) LC–MS/MS identification of stool L‐arginine between PBS and Amuc_1100 groups in vivo. (d) PCA showed variations of gut microbiota composition between two groups, and each character represented a sample. (e) Significant bacterial differences at Genus level between two groups. (f) Representative images of H&E staining in the colon of PBS and Amuc_1100 treated aged mice. Scale bar, 200 μm. (g) Score of inflammation of two groups (*p =* 0.015) (*n* = 5 mice for the PBS group; *n* = 7 mice for the Amuc_1100 group). (h) Relative mRNA levels of *Tnf α* (*p =* 0.0276), *IL 1β* (*p =* 0.0442) and *IL6* gene in colon tissues. (i) Representative immunofluorescence image of ZO‐1 and Ocln in the colon from indicated mice. Scale bar, 100 μm. (j) Relative mRNA levels of *ZO‐1* (*p =* 0.0272) and *Ocln* gene in colon tissues. (k) Plasma LPS level in two groups (*p =* 0.0055). Data were represented as mean ± SD. Comparisons were performed by unpaired two‐tailed *t* test or nonparametric Mann–Whitney test (c, g, h, j and k). *p < 0.05, **p < 0.01; ns, not significant.

To investigate the cause of the elevated plasma L‐arginine due to the treatment of Amuc_1100, we first analyzed the structural composition of Amuc_1100. We found Amuc_1100 contained a certain amount of L‐arginine (Figure 5a). Using an in vitro digestion model, we found that Amuc_1100 could directly produce L‐arginine in artificial intestinal fluid instead of artificial gastric fluid (Figure 5b). Then we further examined the fecal level of L‐arginine in aged mice. Notably, Amuc_1100 treatment increased the fecal level of L‐arginine compared with the control group (Figure 5c). And it was reported that intestinal microorganisms are important source of L‐arginine (Xu et al., 2007). We further performed fecal 16S rRNA sequencing on aged and Amuc_1100 treated aged mice groups. PCA revealed that the composition of the gut microbiota changed sharply in two groups (Figure 5d). And we observed that the abundance of *Bifidobacterium* genus increased in Amuc_1100 treated aged mice (Figure 5e), which was reported to contribute to the L‐arginine production (Xiao et al., 2021). Together, these results suggest that Amuc_1100 acted directly as a source of L‐arginine and modulated the gut microbiota to increase the level of L‐arginine in plasma.

The integrity of gut barrier also plays an important role in the function of nervous system (Thangaleela et al., 2022). As we have observed in young and aged mice, aged mice showed impairment of intestinal barrier (Figure S7a–c) and higher LPS level in plasma (Figure S7d). We then investigated whether Amuc_1100 was involved in the protective effects of the intestinal barrier against aging. Amuc_1100 treated aged mice showed lower inflammation scoring and inflammatory factor level in colon (Figure 5f–h). Besides, the tight junction proteins also increased in the colon of Amuc_1100 treated aged mice (Figure 5i,j), along with lower level of LPS (Figure 5k). The above findings suggested Amuc_1100 maintained intestinal homeostasis in aged mice.

### 3.6 L‐arginine improved intestinal barriers and intestinal cell stemness

**FIGURE 6:**
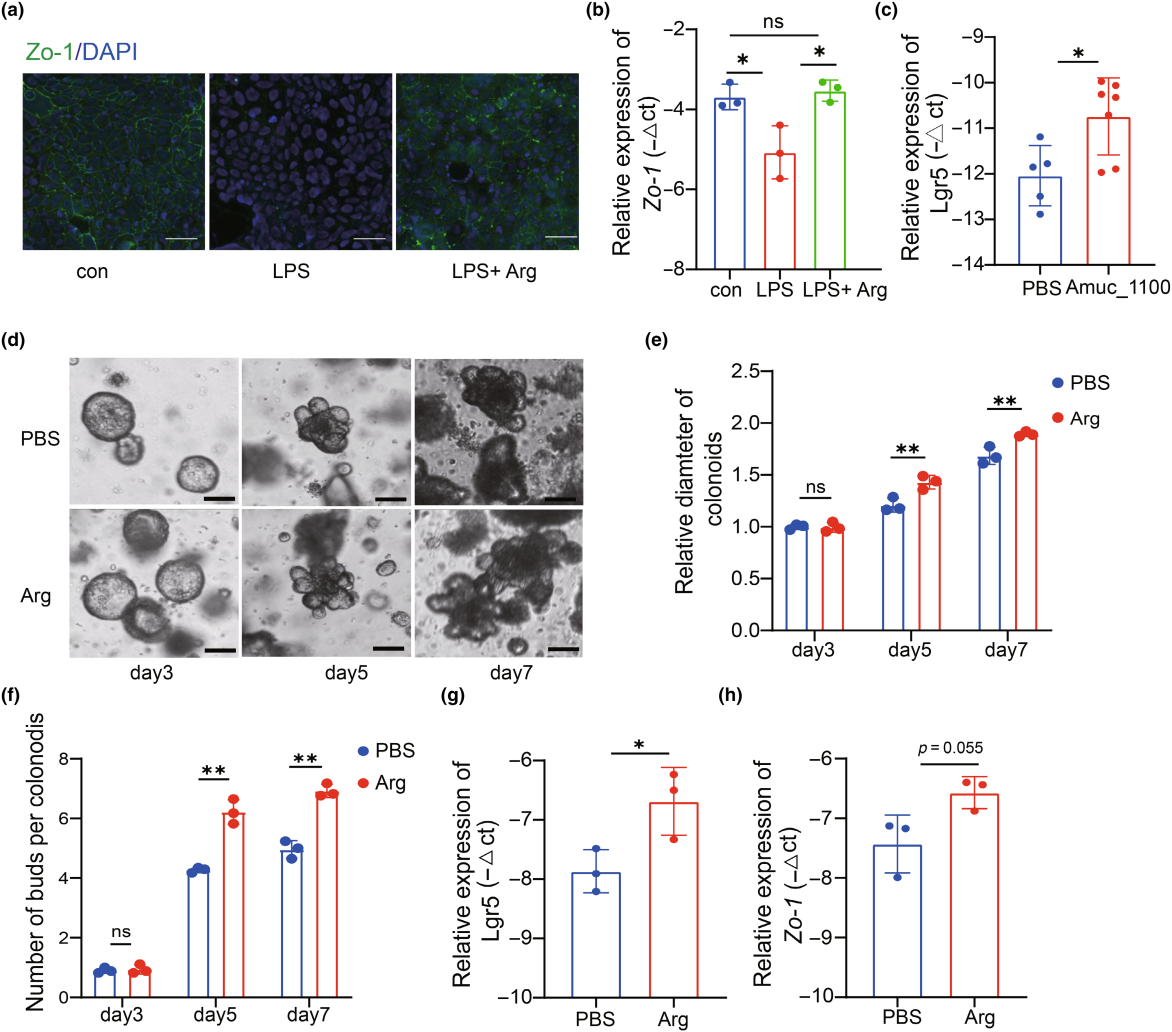
L‐arginine improved intestinal barriers and intestinal cell stemness. (a) Representative immunofluorescence image of ZO‐1 in the Caco2 cell, Scale bar, 50 μm. (*n* = 3 wells per group). (b) Relative mRNA levels of *ZO‐1* (*n* = 3 wells per group). (c) Relative mRNA levels of *Lgr5* gene in the colon of PBS and Amuc_1100 treated aged mice (*p =* 0.0171). (d) Representative images in the organoid‐forming capacity of crypts from control and arginine group. Scale bar, 50 μm. (e) Size of colonoids was quantified and represented relative to untreated control (*n* = 3 wells per group) on day 3, 5 and 7. (f) Comparison de novo buds' number in colonoids (*n* = 3 wells per group). (g) Relative mRNA levels of *Lgr5* gene (*p =* 0.0395). (h) Relative mRNA levels of *Zo1* gene (*p =* 0.055). Data were represented as mean ± SD. Comparisons were performed by one‐way analysis of variance with Tukey's multiple comparisons test (b); two‐way analysis of variance with Sidak's multiple comparisons test (e, f); unpaired two‐tailed *t* test (g, h). *p < 0.05, **p < 0.01, and ***p < 0.001; ns, not significant.

Then we used Caco‐2 cells as a cellular model to further explore the protective effect of L‐arginine on intestinal barrier. We found L‐arginine supported endothelial junctions by upregulating levels of Zo‐1, which was downregulated by LPS stimulation (Figure 6a,b). We found the application of Amuc_1100 increased the level of Lgr5 in colon of aged mice in vivo (Figure 6c). To further confirm the proliferation effect in intestinal cell stemness induced by L‐arginine, colonic crypts were isolated and cultured in Matrigel, and L‐arginine was administered on Day 3. Then we captured and calculated the growth parameters of colonoids on Day 3, Day 5 and Day 7 respectively (Figure 6d). Morphologically, the diameter of colonoids was larger and proportion of budding organoids became higher from Day 3 to Day 7 (Figure 6e,f). And the expression of *Lgr5* gene was upregulated by L‐arginine (Figure 6g). Meanwhile, the expression of *Zo‐1* gene showed an increase in treatment of L‐arginine (Figure 6h). These data suggested that L‐arginine might play a role in the protection of intestinal mucosa from inflammatory damage by maintaining the intestinal stem cells.

## 4 DISCUSSION

Among age‐related diseases, including neurodegenerative diseases, the cognitive decline is particularly important, since they have a large impact on healthy longevity and quality of life. The incidence of “age‐related cognitive decline” (ARCD) is 70% higher than that of dementia in healthy aging people (Juan & Adlard, 2019), which is a serious threat to global population aging. Thus, it is necessary to analyze the occurrence and potential mechanism by which healthy aging leads to cognitive decline. Animal models of aging can help elucidate the mechanisms by which aging leads to cognitive decline and be applied to the assessment of the efficacy of anti‐aging medications. Firstly, our research displayed 20‐month‐old natural‐aging mice and 10‐month‐old accelerated aging mice exhibited poor learning and memory abilities in behavior test compared with control mice. Researchers have reported that the learning and memory abilities was closely related to synaptic function and neuronal activity (Vanguilder & Freeman, 2011). Hippocampal neuronal synapses become dysregulated during aging, possibly due to morphological change, gene expression change or neurotransmitter signaling alterations. As reported, older rats exhibited reduced synaptic density of dentate gyrus and CA3 regions in hippocampus (Juan & Adlard, 2019). Older female monkeys have been shown to develop ARCD due to reduced synaptic connections and synaptic strength in the dentate gyrus (Hara et al., 2011). Excitatory synapses lost was also associated with cognitive impairment in monkeys during aging (Petralia et al., 2014). Thus, synaptic damage caused by aging is, at least in part, caused by altered synaptic plasticity, such as dendritic spine growth or contraction, enhanced synaptic transmission, and even the formation of new synapses (Todorova & Blokland, 2017). In our study, 20‐month‐old natural aging mice and 10‐month‐old accelerated aging mice showed a decline in the density of dendritic spines.

Alterations in gut bacterial composition and gut signaling events affected brain function and behavior through a variety of mechanisms. Gut‐derived metabolites could act as neurotransmitters locally and in the brain. The emerging field of “psychobiology” (Bu et al., 2015; Keshavarzian et al., 2015) proposed manipulating these bacteria to regulate the function of gut and brain. Interestingly, dead bacteria and their metabolites, which were called “postbiotics,” still showed physiological activity. As reported, *Lactobacillus plantarum*‐derived postbiotics could ameliorate *Salmonella*‐induced neurological dysfunctions (Wu et al., 2022). Our results revealed that aged mice exhibited alterations in gut microbiota, especially the low *AKK* abundance, consistent with previous reports (Biagi et al., 2010; Grajeda‐Iglesias et al., 2021; Higarza et al., 2021; van der Lugt et al., 2019; Wu et al., 2020). *AKK* outer membranes‐Amuc_1100 supplementation could delay age‐related neurological decline in two types of aging models including natural or accelerated aging mice. Increasing evidence had demonstrated the role of altered L‐arginine metabolism in aging and neurodegeneration (Liu et al., 2014; Mazlan et al., 2017). L‐arginine regulated a variety of functions, such as inflammation, neurogenesis, and redox stress (Yi et al., 2009). We first observed the impairment of L‐arginine metabolism induced by aging. After the application of Amuc_1100, the L‐arginine metabolism was improved, especially the increase of plasma L‐arginine in aged mice. We found the level of L‐arginine was higher in the brain of aged mice, which was consistent with other researches (Boehme et al., 2021; Mazlan et al., 2017; Rushaidhi et al., 2012). While aging activates immunity, arginine catabolism increases dramatically, leading to a further decrease in arginine bioavailability (Kan et al., 2015; Mazlan et al., 2017; Rushaidhi et al., 2012). In our study, Amuc_1100 treatment reduced L‐arginine levels in the hippocampus, and we speculated that the intervention of Amuc_1100 could increase the bioavailability of L‐arginine. L‐arginine was metabolized into many bioactive molecules in a tightly regulated manner in mammals (Morris, 2007). L‐arginine produced NO through nitric oxide synthase (Nos) (Paul & Ekambaram, 2011), including endothelial Nos (eNos), neuronal Nos (nNos), and inducible Nos (iNos) (Ben‐Azu et al., 2019; Zhang et al., 2013). eNos loss contributed to APP amyloid formation processes and cognitive decline (Bergin et al., 2018), nNos played a vital role in synaptic plasticity and learning and memory (Paul & Ekambaram, 2011), while iNos could act as proinflammatory agents (Haj‐Mirzaian et al., 2016). NO was also involved in synaptic plasticity, long‐term potentiation, and memory consolidation (Ben‐Azu et al., 2019). NO deficiency promoted endothelial dysfunction, accelerated amyloid formation and accumulation, reduced synaptic plasticity, activated microglia, and induces neuroinflammation (Katusic & Austin, 2014). Our results revealed that Amuc_1100‐treated aged mice showed higher level of NO and higher expression of Nos3 in the brain. Increasing oxidative stress is observed in the contexts of aging. Evidence showed that the brain is the organ most vulnerable to oxidative damage because of its high oxygen demand and the low proliferative properties of neurons, representing a common feature of the older brain (Kern & Behl, 2009). Oxidative stress leads to the uncoupling of eNOS, thus losing the normal ability to produce NO and generating superoxide, further aggravating oxidative stress damage. We found the application of Amuc_1100 could improve the antioxidant capacity and reduce oxidative stress levels in vivo. In vitro, compared with the control group, primary neurons co‐cultured with SNP had more SYN‐1 positive spots and exhibited improved synaptic plasticity by improvement of the antioxidant capacity. These revealed the positive regulation of L‐arginine/NO pathway by Amuc_1100 in the brain.

L‐arginine is mainly derived from dietary intake and protein degradation, which participates in protein synthesis and energy metabolism for the host. Using in vitro digestion models, we verified that Amuc_1100 could directly produce L‐arginine in artificial intestinal fluid. The gut microbiota produce dozens of metabolites that accumulate in the bloodstream (Nicholson et al., 2012). We found that the intestinal L‐arginine level was increased in aged mice treated with Amuc_1100 in vivo, which accounted for the high level of L‐arginine in plasma. Meanwhile, intestinal microorganisms were another important source of L‐arginine (Xu et al., 2007), and we found the composition of the gut microbiota was reshaped by Amuc_1100 in aged mice. The *Bifidobacterium* genus, L‐arginine‐producing bacteria, increased in Amuc_1100‐treated aged mice (Xiao et al., 2021). In particular, *B. longum* has been reported to enhance bacterial arginine enrichment, ultimately affecting the overall metabolome of the intestinal microbiota and protecting the host from aging. Thus, both Amuc_1100 and gut microbiota might be the major triggers of a blood increase in L‐arginine. And the application of Amuc_1100 also provides new clues and ideas for the treatment of diseases related to low L‐arginine levels diseases.

The taxonomically diverse gut microbiota have been implicated in the integrity of epithelial barrier and the maintenance of intestinal metabolic homeostasis, as well as the brain function, including neuroinflammation and behavioral diseases (Fung et al., 2017). For example, the mice absence of gut microbiota (such as germ‐free mice), exhibited impaired hippocampal neurogenesis (Erny et al., 2015; Sharon et al., 2016), while transplanting the microbiota of high‐fat donors into healthy mice disrupted the intestinal barrier and caused cognitive decline (Bruce‐Keller et al., 2015; Shi et al., 2021). Gut dysbiosis was often associated with leaky gut. Some postbiotics were reported to enhance the intestinal barrier function, such as exopolysaccharides from *Bifidobacterium* spp. (Schiavi et al., 2016). Our results suggested that *AKK* outer membranes‐ Amuc_1100 also reduced intestinal barrier damage in aged mice, expressed as the increase of tight junction proteins and Lgr5 in the colon. Amuc_1100‐induced L‐arginine elevation has also been shown to maintain intestinal stemness and protect the intestinal barrier in vitro. Moreover, increased intestinal permeability induced bacterial LPS entered the blood circulation (Zhang et al., 2019), which could promote a chronic systemic inflammatory state, leading to age‐related chronic neurodegenerative diseases (Currais, 2015; Hou et al., 2019). Our results found that Amuc_1100 treated aged mice showed lower LPS level in plasma in aged mice. Therefore, it could be speculated that enhancement of intestinal barrier function and reduction of LPS translocation may be crucial for Amuc_1100 to maintain intestinal homeostasis in aging mice.

In future applications, postbiotics is a promising microbiota‐based treatment approaches with avoidance of viable bacterial translocation and more precise effect on epithelial cells. Furthermore, postbiotics is easier to produce, transport and store, providing them with more advantages in clinical application (Salminen et al., 2021). Amuc_1100 was reported to be a novel source for many diseases in future therapies, such as intestinal barrier impairment, inflammatory bowel disease, and type 1 diabetes (Cani & de Vos, 2017). In our study, we uncovered that Amuc_1100 ameliorated age‐related cognition functional decline by maintenance of intestinal homeostasis through the L‐arginine metabolism. This reminds us that the Amuc_1100 and its intermediate metabolites might provide effective and easily manipulated targets for the future treatment of age‐related cognitive impairment. But more research is worthwhile to bring this therapy to the clinic.

## AUTHOR CONTRIBUTIONS

Shujie Chen, Liangjing Wang, and Jianmin Si conceived the project and designed experiments. Jiamin He, Tongyao Hou, Qiwen Wang, Qingyi Wang, Yao Jiang, Luyi Chen, and Jilei Xu performed experiments. Yadong Qi, Dingjiacheng Jia, Yanrou Gu, and Lidan Gao, assisted in the experiment. Lan Wang, Lijun Kang, and Yingcong Yu advised on manuscript preparation. Jiamin He, Tongyao Hou, and Qiwen Wang wrote the manuscript. Shujie Chen, Liangjing Wang, and Jianmin Si reviewed the manuscript. All authors read and approved the final manuscript.

## FUNDING INFORMATION

Provincial Key R&D Program of Zhejiang (2023C03163 to LJ.W.); National Natural Science Foundation of China (82300621 to T.Y H.); National Natural Science Foundation of China (82270573 to SJ.C.); Natural Science Foundation of Zhejiang Province (LQ23H250001 to LY.C.); “Pioneer” and “Leading Goose” R&D Program of Zhejiang (2022C03145 to YC.Y.); Medical Science and Technology Project of Zhejiang Province (2023ky785 to L.W.).

## CONFLICT OF INTEREST STATEMENT

The authors declare that they have no competing interests.

## Supporting information


Data S1.

## Data Availability

All data are available in the main text or the supplementary materials.

## References

[acel14081-bib-0001] Ben‐Azu, B. , Aderibigbe, A. O. , Ajayi, A. M. , Umukoro, S. , & Iwalewa, E. O. (2019). Involvement of l‐arginine‐nitric oxide pathway in the antidepressant and memory promoting effects of morin in mice. Drug Development Research, 80(8), 1071–1079. 10.1002/ddr.21588 31407363

[acel14081-bib-0002] Bergin, D. H. , Jing, Y. , Mockett, B. G. , Zhang, H. , Abraham, W. C. , & Liu, P. (2018). Altered plasma arginine metabolome precedes behavioural and brain arginine metabolomic profile changes in the APPswe/PS1ΔE9 mouse model of Alzheimer's disease. Translational Psychiatry, 8(1), 108. 10.1038/s41398-018-0149-z 29802260 PMC5970225

[acel14081-bib-0003] Biagi, E. , Nylund, L. , Candela, M. , Ostan, R. , Bucci, L. , Pini, E. , Nikkïla, J. , Monti, D. , Satokari, R. , Franceschi, C. , Brigidi, P. , & de Vos, W. (2010). Through ageing, and beyond: Gut microbiota and inflammatory status in seniors and centenarians. PLoS One, 5(5), e10667. 10.1371/journal.pone.0010667 20498852 PMC2871786

[acel14081-bib-0004] Bieri, G. , Schroer, A. B. , & Villeda, S. A. (2023). Blood‐to‐brain communication in aging and rejuvenation. Nature Neuroscience, 26, 379–393. 10.1038/s41593-022-01238-8 36646876

[acel14081-bib-0005] Boehme, M. , Guzzetta, K. E. , Bastiaanssen, T. F. S. , van de Wouw, M. , Moloney, G. M. , Gual‐Grau, A. , Spichak, S. , Olavarría‐Ramírez, L. , Fitzgerald, P. , Morillas, E. , Ritz, N. L. , Jaggar, M. , Cowan, C. S. M. , Crispie, F. , Donoso, F. , Halitzki, E. , Neto, M. C. , Sichetti, M. , Golubeva, A. V. , … Cryan, J. F. (2021). Microbiota from young mice counteracts selective age‐associated behavioral deficits. Nature Aging, 1(8), 666–676. 10.1038/s43587-021-00093-9 37117767

[acel14081-bib-0006] Bruce‐Keller, A. J. , Salbaum, J. M. , Luo, M. , Blanchard, E., 4th , Taylor, C. M. , Welsh, D. A. , & Berthoud, H. R. (2015). Obese‐type gut microbiota induce neurobehavioral changes in the absence of obesity. Biological Psychiatry, 77(7), 607–615. 10.1016/j.biopsych.2014.07.012 25173628 PMC4297748

[acel14081-bib-0007] Bu, X. L. , Yao, X. Q. , Jiao, S. S. , Zeng, F. , Liu, Y. H. , Xiang, Y. , Liang, C. R. , Wang, Q. H. , Wang, X. , Cao, H. Y. , Yi, X. , Deng, B. , Liu, C. H. , Xu, J. , Zhang, L. L. , Gao, C. Y. , Xu, Z. Q. , Zhang, M. , Wang, L. , … Wang, Y. J. (2015). A study on the association between infectious burden and Alzheimer's disease. European Journal of Neurology, 22(12), 1519–1525. 10.1111/ene.12477 24910016

[acel14081-bib-0008] Cani, P. D. , & de Vos, W. M. (2017). Next‐generation beneficial microbes: The case of *Akkermansia muciniphila* . Frontiers in Microbiology, 8, 1765. 10.3389/fmicb.2017.01765 29018410 PMC5614963

[acel14081-bib-0009] Cheng, R. , Xu, W. , Wang, J. , Tang, Z. , & Zhang, M. (2021). The outer membrane protein Amuc_1100 of *Akkermansia muciniphila* alleviates the depression‐like behavior of depressed mice induced by chronic stress. Biochemical and Biophysical Research Communications, 566, 170–176. 10.1016/j.bbrc.2021.06.018 34129964

[acel14081-bib-0010] Currais, A. (2015). Ageing and inflammation—A central role for mitochondria in brain health and disease. Ageing Research Reviews, 21, 30–42. 10.1016/j.arr.2015.02.001 25684584

[acel14081-bib-0011] Erny, D. , Hrabě de Angelis, A. L. , Jaitin, D. , Wieghofer, P. , Staszewski, O. , David, E. , Keren‐Shaul, H. , Mahlakoiv, T. , Jakobshagen, K. , Buch, T. , Schwierzeck, V. , Utermöhlen, O. , Chun, E. , Garrett, W. S. , McCoy, K. , Diefenbach, A. , Staeheli, P. , Stecher, B. , Amit, I. , & Prinz, M. (2015). Host microbiota constantly control maturation and function of microglia in the CNS. Nature Neuroscience, 18(7), 965–977. 10.1038/nn.4030 26030851 PMC5528863

[acel14081-bib-0012] Fung, T. C. , Olson, C. A. , & Hsiao, E. Y. (2017). Interactions between the microbiota, immune and nervous systems in health and disease. Nature Neuroscience, 20(2), 145–155. 10.1038/nn.4476 28092661 PMC6960010

[acel14081-bib-0013] Grajeda‐Iglesias, C. , Durand, S. , Daillère, R. , Iribarren, K. , Lemaitre, F. , Derosa, L. , Aprahamian, F. , Bossut, N. , Nirmalathasan, N. , Madeo, F. , Zitvogel, L. , & Kroemer, G. (2021). Oral administration of *Akkermansia muciniphila* elevates systemic antiaging and anticancer metabolites. Aging (Albany NY), 13(5), 6375–6405. 10.18632/aging.202739 33653967 PMC7993698

[acel14081-bib-0014] Gupta, S. , Mullish, B. H. , & Allegretti, J. R. (2021). Fecal microbiota transplantation: The evolving risk landscape. The American Journal of Gastroenterology, 116(4), 647–656. 10.14309/ajg.0000000000001075 33982930

[acel14081-bib-0015] Haj‐Mirzaian, A. , Amiri, S. , Amini‐Khoei, H. , Rahimi‐Balaei, M. , Kordjazy, N. , Olson, C. O. , Rastegar, M. , Naserzadeh, P. , Marzban, H. , Dehpour, A. R. , Hosseini, M. J. , Samiei, E. , & Mehr, S. E. (2016). Attenuation of oxidative and nitrosative stress in cortical area associates with antidepressant‐like effects of tropisetron in male mice following social isolation stress. Brain Research Bulletin, 124, 150–163. 10.1016/j.brainresbull.2016.04.018 27129671

[acel14081-bib-0016] Hara, Y. , Park, C. S. , Janssen, W. G. , Punsoni, M. , Rapp, P. R. , & Morrison, J. H. (2011). Synaptic characteristics of dentate gyrus axonal boutons and their relationships with aging, menopause, and memory in female rhesus monkeys. Journal of Neuroscience, 31(21), 7737–7744. 10.1523/jneurosci.0822-11.2011 21613486 PMC3103072

[acel14081-bib-0017] Higarza, S. G. , Arboleya, S. , Arias, J. L. , Gueimonde, M. , & Arias, N. (2021). *Akkermansia muciniphila* and environmental enrichment reverse cognitive impairment associated with high‐fat high‐cholesterol consumption in rats. Gut Microbes, 13(1), 1–20. 10.1080/19490976.2021.1880240 PMC7946069 33678110

[acel14081-bib-0018] Hou, Y. , Dan, X. , Babbar, M. , Wei, Y. , Hasselbalch, S. G. , Croteau, D. L. , & Bohr, V. A. (2019). Ageing as a risk factor for neurodegenerative disease. Nature Reviews. Neurology, 15(10), 565–581. 10.1038/s41582-019-0244-7 31501588

[acel14081-bib-0019] Juan, S. M. A. , & Adlard, P. A. (2019). Ageing and cognition. Sub‐Cellular Biochemistry, 91, 107–122. 10.1007/978-981-13-3681-2_5 30888651

[acel14081-bib-0020] Kan, M. J. , Lee, J. E. , Wilson, J. G. , Everhart, A. L. , Brown, C. M. , Hoofnagle, A. N. , Jansen, M. , Vitek, M. P. , Gunn, M. D. , & Colton, C. A. (2015). Arginine deprivation and immune suppression in a mouse model of Alzheimer's disease. Journal of Neuroscience, 35(15), 5969–5982. 10.1523/jneurosci.4668-14.2015 25878270 PMC4397598

[acel14081-bib-0021] Katusic, Z. S. , & Austin, S. A. (2014). Endothelial nitric oxide: Protector of a healthy mind. European Heart Journal, 35(14), 888–894. 10.1093/eurheartj/eht544 24357508 PMC3977136

[acel14081-bib-0022] Kern, A. , & Behl, C. (2009). The unsolved relationship of brain aging and late‐onset Alzheimer disease. Biochimica et Biophysica Acta, 1790(10), 1124–1132. 10.1016/j.bbagen.2009.07.016 19632303

[acel14081-bib-0023] Keshavarzian, A. , Green, S. J. , Engen, P. A. , Voigt, R. M. , Naqib, A. , Forsyth, C. B. , Mutlu, E. , & Shannon, K. M. (2015). Colonic bacterial composition in Parkinson's disease. Movement Disorders, 30(10), 1351–1360. 10.1002/mds.26307 26179554

[acel14081-bib-0024] Li, G. , Regunathan, S. , Barrow, C. J. , Eshraghi, J. , Cooper, R. , & Reis, D. J. (1994). Agmatine: An endogenous clonidine‐displacing substance in the brain. Science, 263(5149), 966–969. 10.1126/science.7906055 7906055

[acel14081-bib-0025] Li, Z. , Lai, J. , Zhang, P. , Ding, J. , Jiang, J. , Liu, C. , Huang, H. , Zhen, H. , Xi, C. , Sun, Y. , Wu, L. , Wang, L. , Gao, X. , Li, Y. , Fu, Y. , Jie, Z. , Li, S. , Zhang, D. , Chen, Y. , … Hu, S. (2022). Multi‐omics analyses of serum metabolome, gut microbiome and brain function reveal dysregulated microbiota‐gut‐brain axis in bipolar depression. Molecular Psychiatry, 27(10), 4123–4135. 10.1038/s41380-022-01569-9 35444255

[acel14081-bib-0026] Ling, Z. , Liu, X. , Cheng, Y. , Yan, X. , & Wu, S. (2022). Gut microbiota and aging. Critical Reviews in Food Science and Nutrition, 62(13), 3509–3534. 10.1080/10408398.2020.1867054 33377391

[acel14081-bib-0027] Liu, P. , Fleete, M. S. , Jing, Y. , Collie, N. D. , Curtis, M. A. , Waldvogel, H. J. , Faull, R. L. , Abraham, W. C. , & Zhang, H. (2014). Altered arginine metabolism in Alzheimer's disease brains. Neurobiology of Aging, 35(9), 1992–2003. 10.1016/j.neurobiolaging.2014.03.013 24746363

[acel14081-bib-0028] Liu, R. , Zhu, Z. , Qian, D. , & Duan, J. A. (2019). Comparison of the peptidome released from keratins in Saiga antelope horn and goat horn under simulated gastrointestinal digestion. Electrophoresis, 40(20), 2759–2766. 10.1002/elps.201900078 31162671

[acel14081-bib-0029] Mao, Y. , Shi, D. , Li, G. , & Jiang, P. (2022). Citrulline depletion by ASS1 is required for proinflammatory macrophage activation and immune responses. Molecular Cell, 82(3), 527–541.e7. 10.1016/j.molcel.2021.12.006 35016033

[acel14081-bib-0030] Mazlan, M. , Hamezah, H. S. , Taridi, N. M. , Jing, Y. , Liu, P. , Zhang, H. , Wan Ngah, W. Z. , & Damanhuri, H. A. (2017). Effects of aging and tocotrienol‐rich fraction supplementation on brain arginine metabolism in rats. Oxidative Medicine and Cellular Longevity, 2017, 6019796. 10.1155/2017/6019796 29348790 PMC5733770

[acel14081-bib-0031] Morris, S. M., Jr. (2007). Arginine metabolism: Boundaries of our knowledge. The Journal of Nutrition, 137(6 Suppl 2), 1602s–1609s. 10.1093/jn/137.6.1602S 17513435

[acel14081-bib-0500] Needham, B. D. , Funabashi, M. , Adame, M. D , Wang, Z. , Boktor, J. C. , Haney, J. , Wu, W. L. , Rabut, C. , Ladinsky, M. S. , Hwang, S. J. , Guo, Y. , Zhu, Q. , Griffiths, J. A. , Knight, R. , Bjorkman, P. J. , Shapiro, M. G. , Geschwind, D. H. , Holschneider, D. P. , Fischbach, M. A. , & Mazmanian, S. K. (2022). A gut‐derived metabolite alters brain activity and anxiety behaviour in mice. Nature, 602(7898), 647–653. PMID: 35165440. 10.1038/s41586-022-04396-8 35165440 PMC9170029

[acel14081-bib-0032] Nicholson, J. K. , Holmes, E. , & Elliott, P. (2008). The metabolome‐wide association study: A new look at human disease risk factors. Journal of Proteome Research, 7(9), 3637–3638. 10.1021/pr8005099 18707153

[acel14081-bib-0033] Nicholson, J. K. , Holmes, E. , Kinross, J. , Burcelin, R. , Gibson, G. , Jia, W. , & Pettersson, S. (2012). Host‐gut microbiota metabolic interactions. Science, 336(6086), 1262–1267. 10.1126/science.1223813 22674330

[acel14081-bib-0034] Parra‐Damas, A. , Chen, M. , Enriquez‐Barreto, L. , Ortega, L. , Acosta, S. , Perna, J. C. , Fullana, M. N. , Aguilera, J. , Rodríguez‐Alvarez, J. , & Saura, C. A. (2017). CRTC1 function during memory encoding is disrupted in neurodegeneration. Biological Psychiatry, 81(2), 111–123. 10.1016/j.biopsych.2016.06.025 27587263

[acel14081-bib-0035] Paul, V. , & Ekambaram, P. (2011). Involvement of nitric oxide in learning & memory processes. The Indian Journal of Medical Research, 133(5), 471–478. 21623030 PMC3121276

[acel14081-bib-0036] Petralia, R. S. , Mattson, M. P. , & Yao, P. J. (2014). Communication breakdown: The impact of ageing on synapse structure. Ageing Research Reviews, 14, 31–42. 10.1016/j.arr.2014.01.003 24495392 PMC4094371

[acel14081-bib-0037] Plovier, H. , Everard, A. , Druart, C. , Depommier, C. , van Hul, M. , Geurts, L. , Chilloux, J. , Ottman, N. , Duparc, T. , Lichtenstein, L. , Myridakis, A. , Delzenne, N. M. , Klievink, J. , Bhattacharjee, A. , van der Ark, K. , Aalvink, S. , Martinez, L. O. , Dumas, M. E. , Maiter, D. , … Cani, P. D. (2017). A purified membrane protein from *Akkermansia muciniphila* or the pasteurized bacterium improves metabolism in obese and diabetic mice. Nature Medicine, 23(1), 107–113. 10.1038/nm.4236 27892954

[acel14081-bib-0038] Qi, Y. , He, J. , Zhang, Y. , Ge, Q. , Wang, Q. , Chen, L. , Xu, J. , Wang, L. , Chen, X. , Jia, D. , Lin, Y. , Xu, C. , Zhang, Y. , Hou, T. , Si, J. , Chen, S. , & Wang, L. (2023). Heat‐inactivated *Bifidobacterium adolescentis* ameliorates colon senescence through Paneth‐like‐cell‐mediated stem cell activation. Nature Communications, 14(1), 6121. 10.1038/s41467-023-41827-0 PMC10542354 37777508

[acel14081-bib-0039] Rushaidhi, M. , Jing, Y. , Kennard, J. T. , Collie, N. D. , Williams, J. M. , Zhang, H. , & Liu, P. (2012). Aging affects L‐arginine and its metabolites in memory‐associated brain structures at the tissue and synaptoneurosome levels. Neuroscience, 209, 21–31. 10.1016/j.neuroscience.2012.02.021 22387109

[acel14081-bib-0040] Salminen, S. , Collado, M. C. , Endo, A. , Hill, C. , Lebeer, S. , Quigley, E. M. M. , Sanders, M. E. , Shamir, R. , Swann, J. R. , Szajewska, H. , & Vinderola, G. (2021). The International Scientific Association of Probiotics and Prebiotics (ISAPP) consensus statement on the definition and scope of postbiotics. Nature Reviews Gastroenterology & Hepatology, 18(9), 649–667. 10.1038/s41575-021-00440-6 33948025 PMC8387231

[acel14081-bib-0041] Schiavi, E. , Gleinser, M. , Molloy, E. , Groeger, D. , Frei, R. , Ferstl, R. , Rodriguez‐Perez, N. , Ziegler, M. , Grant, R. , Moriarty, T. F. , Plattner, S. , Healy, S. , O'Connell Motherway, M. , Akdis, C. A. , Roper, J. , Altmann, F. , van Sinderen, D. , & O'Mahony, L. (2016). The surface‐associated exopolysaccharide of *Bifidobacterium longum* 35624 plays an essential role in dampening host proinflammatory responses and repressing local TH17 responses. Applied and Environmental Microbiology, 82(24), 7185–7196. 10.1128/aem.02238-16 27736791 PMC5118929

[acel14081-bib-0042] Sharon, G. , Sampson, T. R. , Geschwind, D. H. , & Mazmanian, S. K. (2016). The central nervous system and the gut microbiome. Cell, 167(4), 915–932. 10.1016/j.cell.2016.10.027 27814521 PMC5127403

[acel14081-bib-0043] Shi, H. , Ge, X. , Ma, X. , Zheng, M. , Cui, X. , Pan, W. , Zheng, P. , Yang, X. , Zhang, P. , Hu, M. , Hu, T. , Tang, R. , Zheng, K. , Huang, X. F. , & Yu, Y. (2021). A fiber‐deprived diet causes cognitive impairment and hippocampal microglia‐mediated synaptic loss through the gut microbiota and metabolites. Microbiome, 9(1), 223. 10.1186/s40168-021-01172-0 34758889 PMC8582174

[acel14081-bib-0044] Thangaleela, S. , Sivamaruthi, B. S. , Kesika, P. , & Chaiyasut, C. (2022). Role of probiotics and diet in the management of neurological diseases and mood states: A review. Microorganisms, 10(11), 2268. 10.3390/microorganisms10112268 36422338 PMC9696277

[acel14081-bib-0045] Todorova, V. , & Blokland, A. (2017). Mitochondria and synaptic plasticity in the mature and aging nervous system. Current Neuropharmacology, 15(1), 166–173. 10.2174/1570159x14666160414111821 27075203 PMC5327446

[acel14081-bib-0046] van der Lugt, B. , van Beek, A. A. , Aalvink, S. , Meijer, B. , Sovran, B. , Vermeij, W. P. , Brandt, R. M. C. , de Vos, W. M. , Savelkoul, H. F. J. , Steegenga, W. T. , & Belzer, C. (2019). *Akkermansia muciniphila* ameliorates the age‐related decline in colonic mucus thickness and attenuates immune activation in accelerated aging Ercc1 (−/Δ7) mice. Immunity & Ageing, 16, 6. 10.1186/s12979-019-0145-z 30899315 PMC6408808

[acel14081-bib-0047] Vanguilder, H. D. , & Freeman, W. M. (2011). The hippocampal neuroproteome with aging and cognitive decline: Past progress and future directions. Frontiers in Aging Neuroscience, 3, 8. 10.3389/fnagi.2011.00008 21647399 PMC3102218

[acel14081-bib-0048] Velazquez, R. , Ferreira, E. , Knowles, S. , Fux, C. , Rodin, A. , Winslow, W. , & Oddo, S. (2019). Lifelong choline supplementation ameliorates Alzheimer's disease pathology and associated cognitive deficits by attenuating microglia activation. Aging Cell, 18(6), e13037. 10.1111/acel.13037 31560162 PMC6826123

[acel14081-bib-0049] Villeda, S. A. , Luo, J. , Mosher, K. I. , Zou, B. , Britschgi, M. , Bieri, G. , Stan, T. M. , Fainberg, N. , Ding, Z. , Eggel, A. , Lucin, K. M. , Czirr, E. , Park, J. S. , Couillard‐Després, S. , Aigner, L. , Li, G. , Peskind, E. R. , Kaye, J. A. , Quinn, J. F. , … Wyss‐Coray, T. (2011). The ageing systemic milieu negatively regulates neurogenesis and cognitive function. Nature, 477(7362), 90–94. 10.1038/nature10357 21886162 PMC3170097

[acel14081-bib-0050] Villeda, S. A. , Plambeck, K. E. , Middeldorp, J. , Castellano, J. M. , Mosher, K. I. , Luo, J. , Smith, L. K. , Bieri, G. , Lin, K. , Berdnik, D. , Wabl, R. , Udeochu, J. , Wheatley, E. G. , Zou, B. , Simmons, D. A. , Xie, X. S. , Longo, F. M. , & Wyss‐Coray, T. (2014). Young blood reverses age‐related impairments in cognitive function and synaptic plasticity in mice. Nature Medicine, 20(6), 659–663. 10.1038/nm.3569 PMC4224436 24793238

[acel14081-bib-0051] Wang, L. , Tang, L. , Feng, Y. , Zhao, S. , Han, M. , Zhang, C. , Yuan, G. , Zhu, J. , Cao, S. , Wu, Q. , Li, L. , & Zhang, Z. (2020). A purified membrane protein from *Akkermansia muciniphila* or the pasteurised bacterium blunts colitis associated tumourigenesis by modulation of CD8(+) T cells in mice. Gut, 69(11), 1988–1997. 10.1136/gutjnl-2019-320105 32169907 PMC7569398

[acel14081-bib-0052] Wu, F. , Guo, X. , Zhang, M. , Ou, Z. , Wu, D. , Deng, L. , Lu, Z. , Zhang, J. , Deng, G. , Chen, S. , Li, S. , Yi, J. , & Peng, Y. (2020). An *Akkermansia muciniphila* subtype alleviates high‐fat diet‐induced metabolic disorders and inhibits the neurodegenerative process in mice. Anaerobe, 61, 102138. 10.1016/j.anaerobe.2019.102138 31830598

[acel14081-bib-0053] Wu, G. , & Morris, S. M., Jr. (1998). Arginine metabolism: Nitric oxide and beyond. The Biochemical Journal, 336(Pt 1), 1–17. 10.1042/bj3360001 9806879 PMC1219836

[acel14081-bib-0054] Wu, Y. , Wang, Y. , Hu, A. , Shu, X. , Huang, W. , Liu, J. , Wang, B. , Zhang, R. , Yue, M. , & Yang, C. (2022). *Lactobacillus plantarum*‐derived postbiotics prevent *Salmonella*‐induced neurological dysfunctions by modulating gut‐brain axis in mice. Frontiers in Nutrition, 9, 946096. 10.3389/fnut.2022.946096 35967771 PMC9365972

[acel14081-bib-0055] Wyss‐Coray, T. (2016). Ageing, neurodegeneration and brain rejuvenation. Nature, 539(7628), 180–186. 10.1038/nature20411 27830812 PMC5172605

[acel14081-bib-0056] Xiao, Y. , Yang, C. , Yu, L. , Tian, F. , Wu, Y. , Zhao, J. , Zhang, H. , Yang, R. , Chen, W. , Hill, C. , Cui, Y. , & Zhai, Q. (2021). Human gut‐derived *B. longum* subsp. *longum* strains protect against aging in a D‐galactose‐induced aging mouse model. Microbiome, 9(1), 180. 10.1186/s40168-021-01108-8 34470652 PMC8411540

[acel14081-bib-0057] Xie, K. , Qin, Q. , Long, Z. , Yang, Y. , Peng, C. , Xi, C. , Li, L. , Wu, Z. , Daria, V. , Zhao, Y. , Wang, F. , & Wang, M. (2021). High‐throughput metabolomics for discovering potential biomarkers and identifying metabolic mechanisms in aging and Alzheimer's disease. Frontiers in Cell and Developmental Biology, 9, 602887. 10.3389/fcell.2021.602887 33718349 PMC7947003

[acel14081-bib-0058] Xu, Y. , Labedan, B. , & Glansdorff, N. (2007). Surprising arginine biosynthesis: A reappraisal of the enzymology and evolution of the pathway in microorganisms. Microbiology and Molecular Biology Reviews, 71(1), 36–47. 10.1128/mmbr.00032-06 17347518 PMC1847373

[acel14081-bib-0059] Yi, J. , Horky, L. L. , Friedlich, A. L. , Shi, Y. , Rogers, J. T. , & Huang, X. (2009). L‐arginine and Alzheimer's disease. International Journal of Clinical and Experimental Pathology, 2(3), 211–238. 19079617 PMC2600464

[acel14081-bib-0060] Zhang, F. L. , Yang, Y. L. , Zhang, Z. , Yao, Y. Y. , Xia, R. , Gao, C. C. , du, D. D. , Hu, J. , Ran, C. , Liu, Z. , & Zhou, Z. G. (2021). Surface‐displayed Amuc_1100 from *Akkermansia muciniphila* on *Lactococcus lactis* ZHY1 improves hepatic steatosis and intestinal health in high‐fat‐fed zebrafish. Frontiers in Nutrition, 8, 726108. 10.3389/fnut.2021.726108 34722607 PMC8548614

[acel14081-bib-0061] Zhang, G. F. , Wang, N. , Shi, J. Y. , Xu, S. X. , Li, X. M. , Ji, M. H. , Zuo, Z. Y. , Zhou, Z. Q. , & Yang, J. J. (2013). Inhibition of the L‐arginine‐nitric oxide pathway mediates the antidepressant effects of ketamine in rats in the forced swimming test. Pharmacology Biochemistry and Behavior, 110, 8–12. 10.1016/j.pbb.2013.05.010 23711590

[acel14081-bib-0062] Zhang, P. , Yu, Y. , Qin, Y. , Zhou, Y. , Tang, R. , Wang, Q. , Li, X. , Wang, H. , Weston‐Green, K. , Huang, X. F. , & Zheng, K. (2019). Alterations to the microbiota‐colon‐brain axis in high‐fat‐diet‐induced obese mice compared to diet‐resistant mice. The Journal of Nutritional Biochemistry, 65, 54–65. 10.1016/j.jnutbio.2018.08.016 30623851

[acel14081-bib-0063] Zhang, Y. , Chen, R. , Zhang, D. , Qi, S. , & Liu, Y. (2023). Metabolite interactions between host and microbiota during health and disease: Which feeds the other? Biomedicine & Pharmacotherapy, 160, 114295. 10.1016/j.biopha.2023.114295 36709600

[acel14081-bib-0600] Zheng,, H. , Xu,, P. , Jiang, Q. , Xu, Q. , Zheng, Y. , Yan, J. , Ji, H. , Ning, J. , Zhang, X. , Li, C. , Zhang, L. , Li, Y. , Li, X. , Song, W. , & Gao, H. (2021). Depletion of acetate‐producing bacteria from the gut microbiota facilitates cognitive impairment through the gut‐brain neural mechanism in diabetic mice. Microbiome, 9(1), 145. PMID: 34172092. 10.1186/s40168-021-01088-9 34172092 PMC8235853

