# Supplementary material for: L‐arginine metabolism ameliorates age‐related cognitive impairment by Amuc_1100‐mediated gut homeostasis maintaining
Source: Aging Cell. 2024 Jan 18;23(4):e14081. doi: 10.1111/acel.14081 (PMC11019123; doi:10.1111/acel.14081)
Supplement: Supplementary file 1 — Data S1. [file ACEL-23-e14081-s001.docx]

**Appendix**

**Supporting Experimental Section**

**Open-field test**

The mouse was placed at the center point of a square open field (50 cm×50 cm×40 cm) and allowed to explore freely for 5 min. All trials were video recorded via the SMART Video Tracking System. The total distances traveled in the central area (central distances) was analyzed. After each test, we wiped the device with 75% alcohol and let it dry to prevent possible odor influences.

**Elevated plus-maze test**

The elevated cross maze consisted of four arms (width, 5 cm; length, 30 cm) comprising two closed (black walls, 15 cm high) and two open (without walls) arms, which are perpendicular to each other and cross. At the beginning of the experiment, each mouse was released at the central point with its head facing the open arm and allowed to explore freely for 5 min. The time spent in the open arms (OA time) and number of entries in the open arms (number of OA entries) were recorded and analyzed. After the completion of the experiment, we wiped the maze with 75% alcohol and let it dry to prevent any odor influence.

**Thickness of the colonic mucus layer**

The thickness of the colonic mucus layer was measured by staining the colonic tissue sections with Muc2. The anti-Muc2 antibody (Cat.GB11344, Servicebio) was diluted 1:500 in TBS and Alexa Fluor® 488 (Cat. GB25303, Servicebio, China) was used for secondary antibody.

**Supporting Results**

**FigureS1**


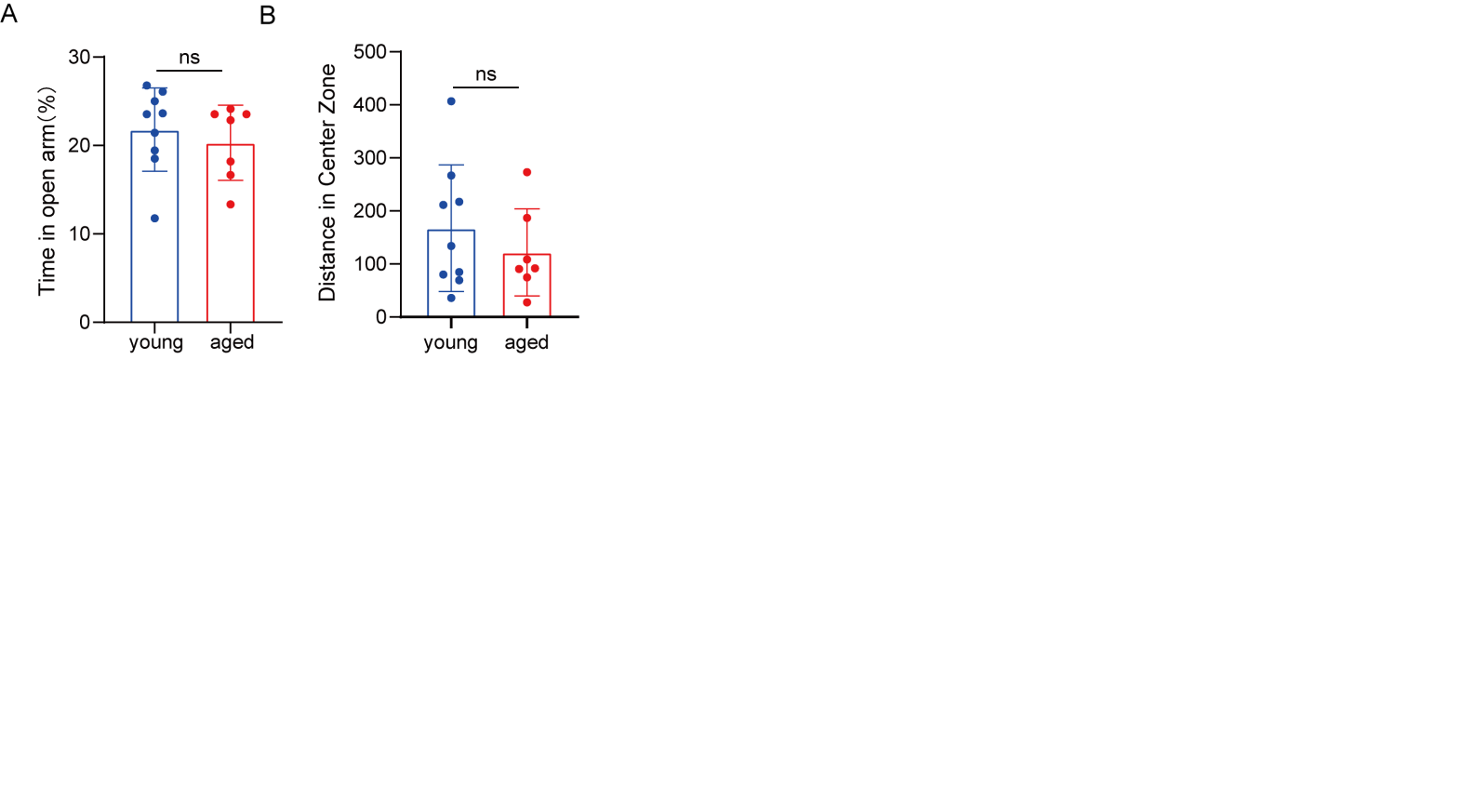


A. The time spent in the open arms (OA time) of two groups in the elevated plus-maze (EPM)test.

B. The central distance of two groups in the Open-field (OFT) test.

Data were represented as mean ± SD. **p*< 0.05, ***p*< 0.01, and ****p*< 0.001; ns, not significant.

**FigureS2**


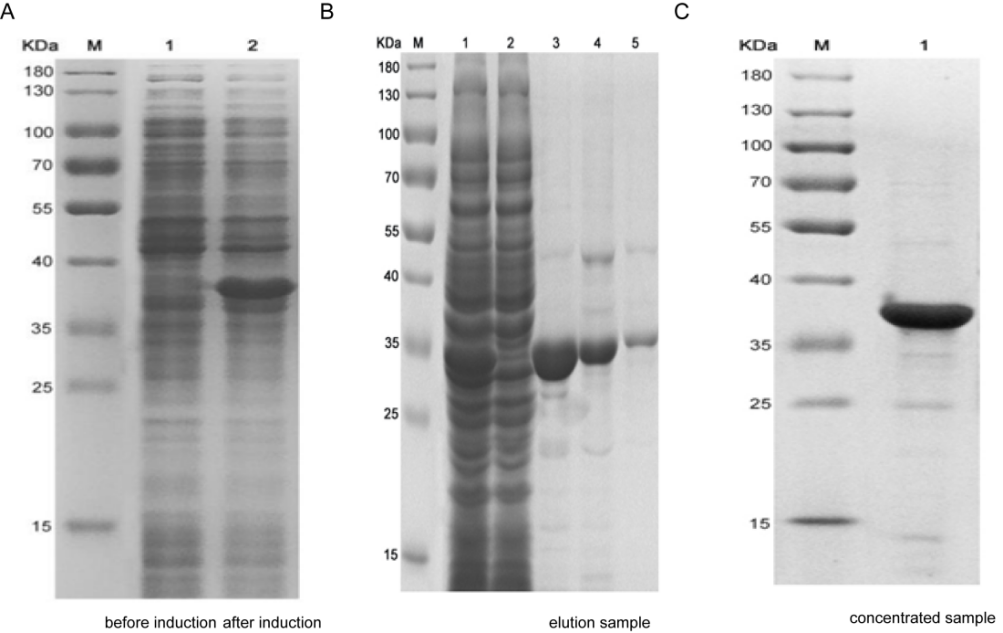


A. SDS-PAGE results of expression test. lane M: maker; lane 1: pre-induction sample; lane 2: post-induction sample.

B. Lane M: marker; lane 1: broken bacteria; lane 2: flow-through solution; lanes 3-5: elution sample.

C. Lane M: marker; Lane 1: concentrated sample.

**FigureS3**


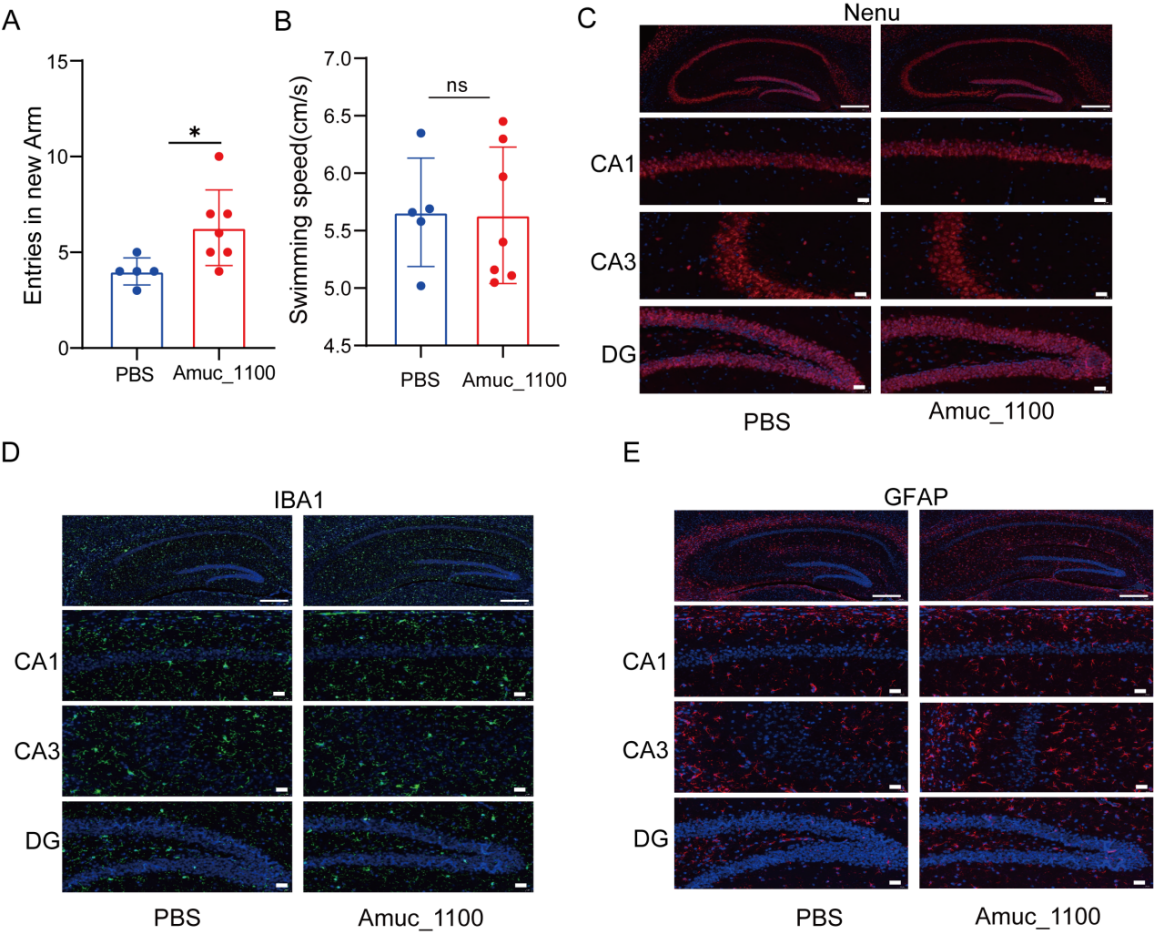


1. In the Y-maze forced alternation test, the entries in new arms (*p*=0.0344) of two groups (n = 5 mice for the PBS group; n = 7 mice for the Amuc_1100 group).
2. Mice mean swimming speed in the probe trial of PBS and Amuc_1100 treated aged mice in the MWM test (n = 5 mice for the PBS group; n = 7 mice for the Amuc_1100 group).
3. Representative images of immunofluorescence staining of NeuN (red) and DAPI (blue) in the hippocampus. Thin scale bars, 100 µm; Thick scale bars, 20 µm;
4. Representative images of immunofluorescence staining of IBA1^+^ cells in hippocampus. Thin scale bars, 100 µm; Thick scale bars, 20 µm;
5. Representative images of immunofluorescence staining of GFAP^+^ cells in hippocampus. Thin scale bars, 100 µm; Thick scale bars, 20 µm;

Data were represented as mean ± SD. **p* < 0.05, ***p*< 0.01, and ****p* < 0.001; ns, not significant.

**FigureS4**

**
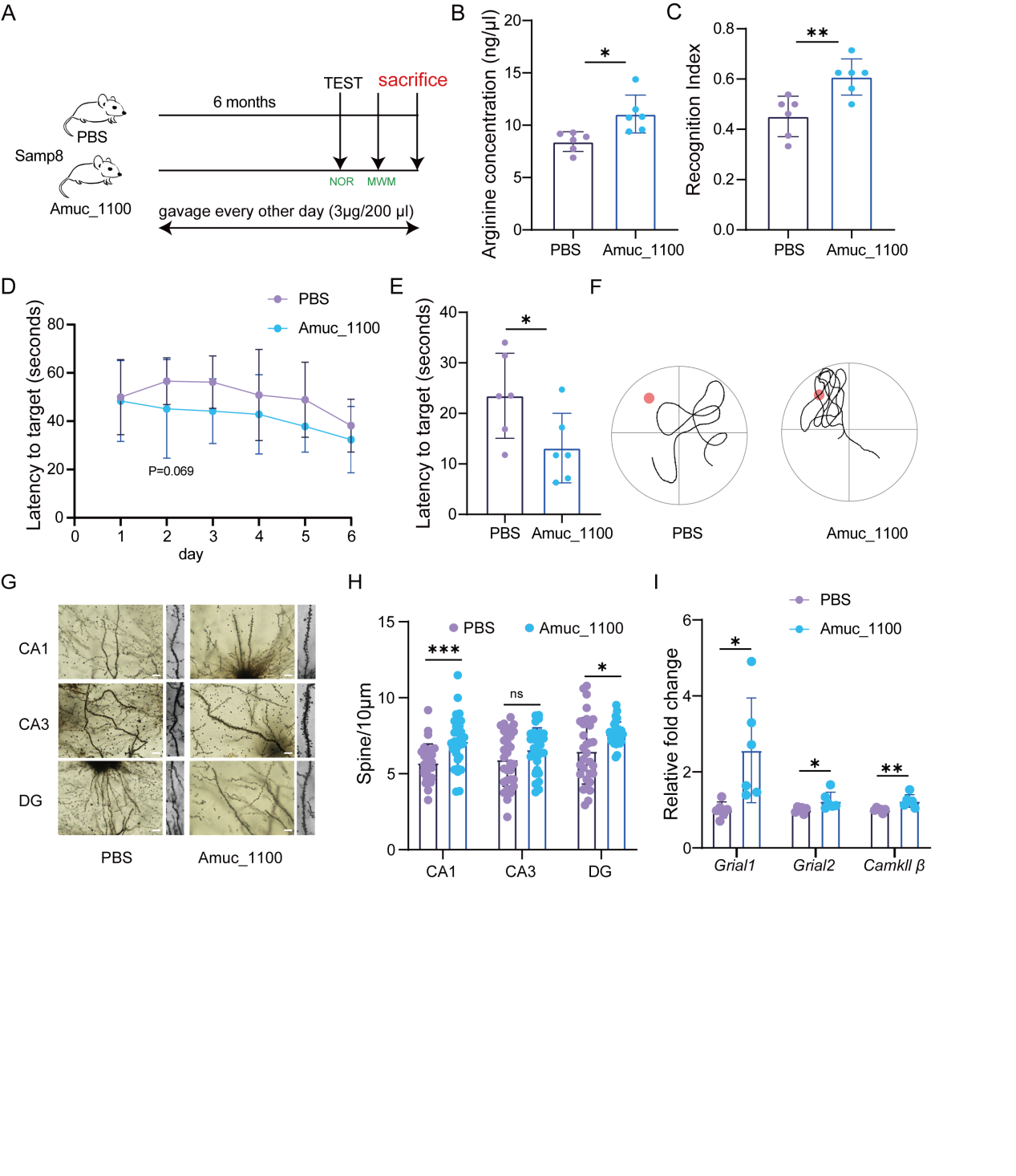
**

1. Schematic diagram showing the strategy in PBS and Amuc_1100 treated aged Samp8 mice.
2. LC-MS/MS identification of plasma L-arginine level between PBS and Amuc_1100 groups in vivo ( n = 6 mice for the Samp8+Amuc_1100 group; n = 6 mice for the Samp8+Amuc_1100 group).
3. Recognition index of mice in NOR test.
4. Latency of first time to enter the target during the training period in a MWM test.
5. Latency of first time to enter the target in the probe trial of the MWM test.
6. Representative trajectory diagram in the probe trial of the MWM test.
7. Representative Golgi-Cox-staining images showing the density and morphology of dendritic spines in the cone cell layer of the murine hippocampal CA1, CA3 and DG region in each group. Scale bar, 10μm.
8. Analysis of total dendritic spine density of the CA1, CA3 and DG region hippocampal region in each group (n = 3 mice for each group, total 180 neurons).
9. Relative mRNA levels of *Grial1*, *Grial2* and *CaMKllβ* gene in hippocampal tissues by RT-PCR. Data were represented as mean ± SD. **p* < 0.05, ***p* < 0.01, and ****p* < 0.001; ns, not significant.

**Figure S5**

**
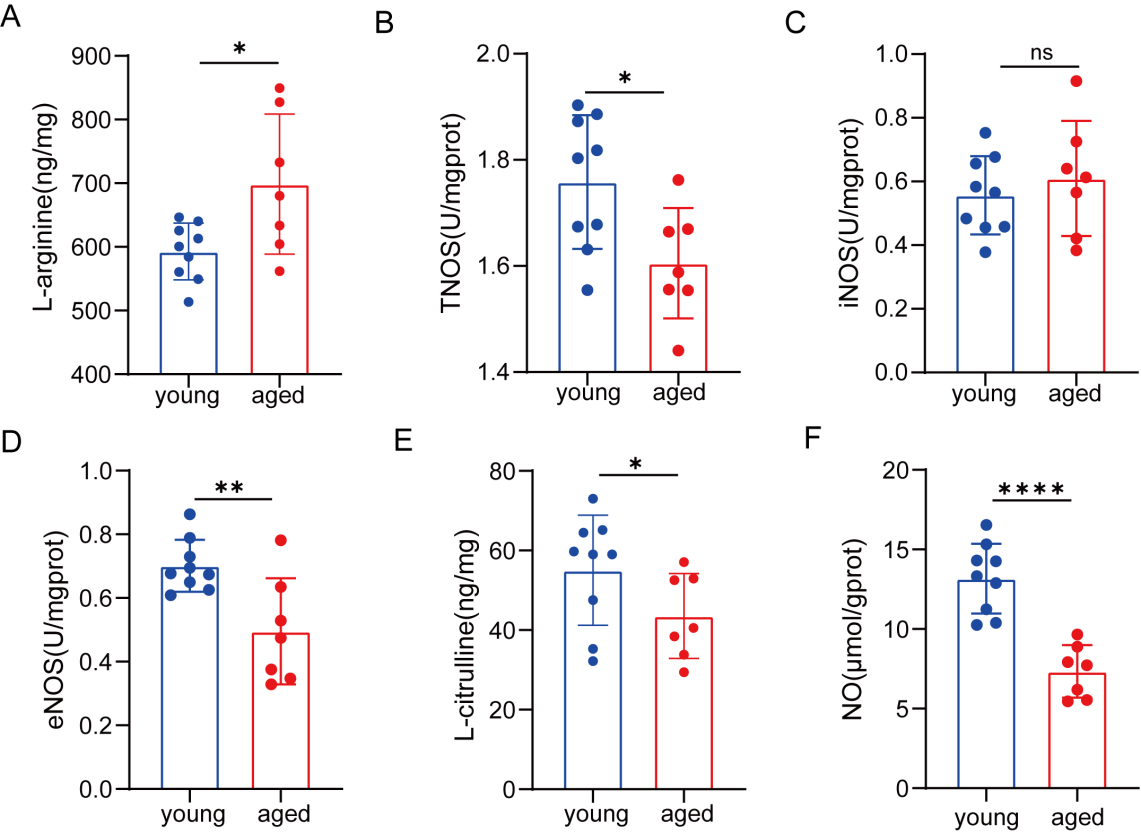
**

1. LC-MS/MS identification of L-arginine level between young and aged groups in hippocampus.

B-D. The activity of NOS enzymes in the hippocampus.

E. LC-MS/MS identification of L-citrulline level between young and aged groups in hippocampus.

F. The level of NO in hippocampus of two groups.

Data were represented as mean ± SD. **p* < 0.05, ***p*< 0.01, and ****p* < 0.001; ns, not significant.

**Figure S6**

**
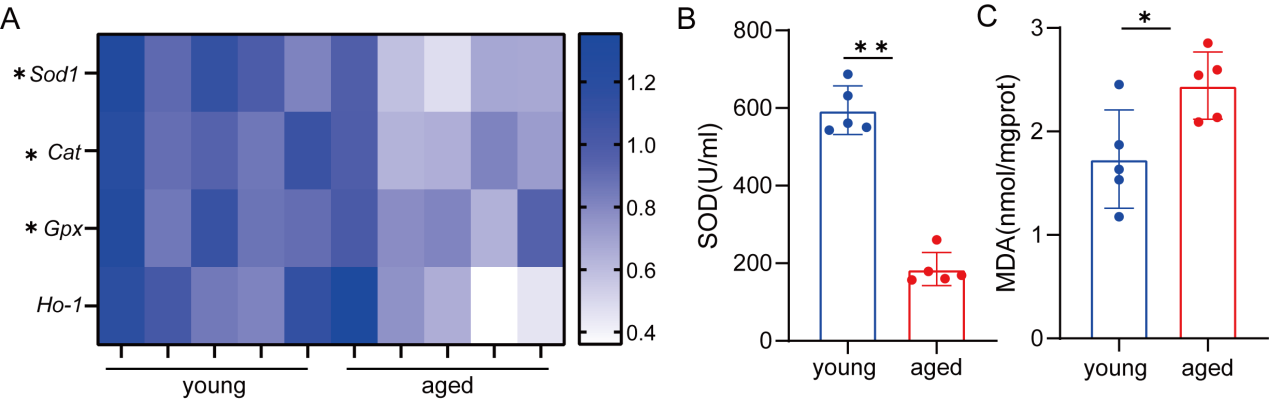
**

A.Relative mRNA levels of *Sod1*, *Cat*, *Gpx* and *Ho-1* gene in the hippocampal tissues of young and aged mice.

B-C.The SOD activity and MDA levels the in the hippocampal of young and aged mice.

Data were represented as mean ± SD. **p* < 0.05, ***p*< 0.01, and ****p* < 0.001; ns, not significant.

**Figure S7**


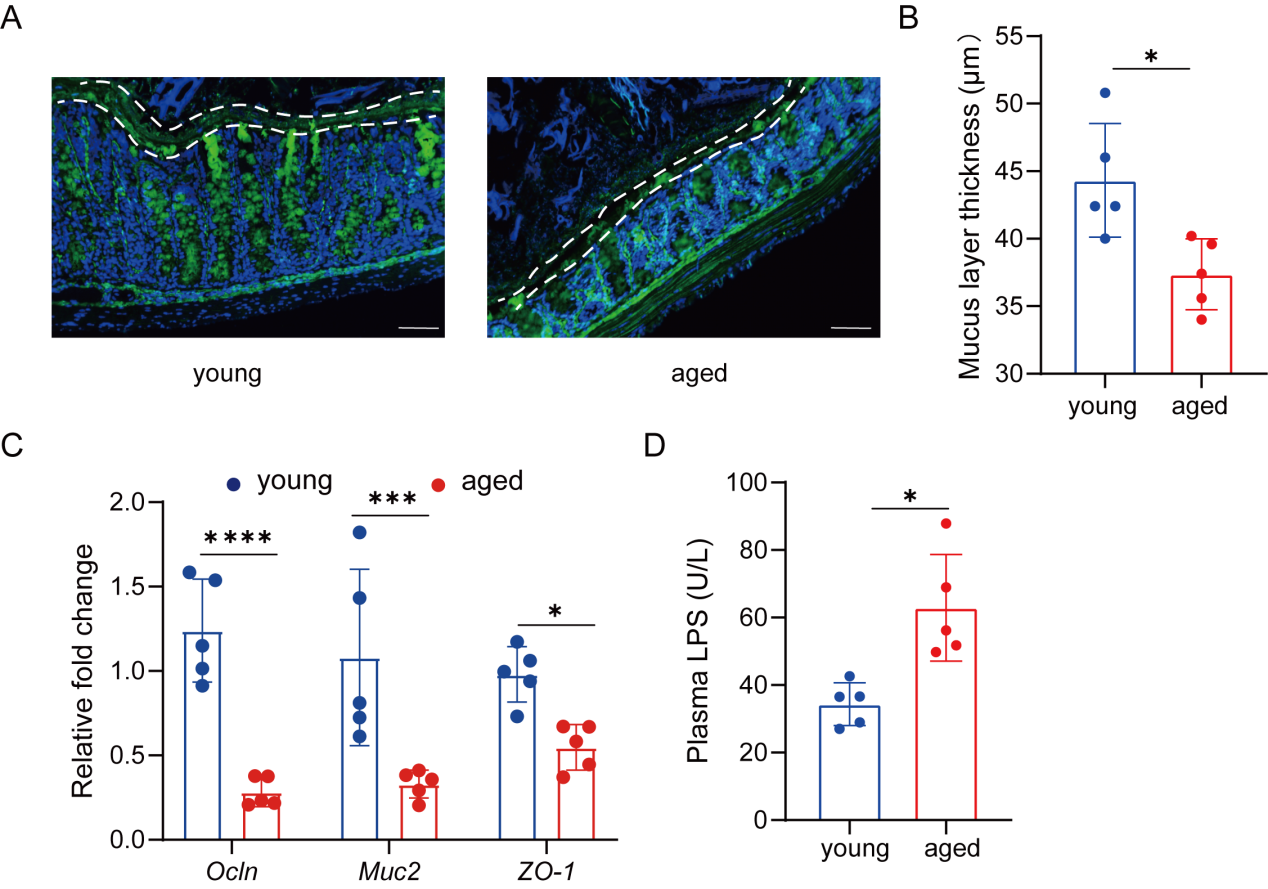


1. Immunofluorescence images of colonic sections stained with Anti-MUC2 antibody and DAPI. Scale bar, 50 μm.
2. The quantification of the colonic mucus layer was statistically analyzed.
3. Relative mRNA levels of *Ocln*, *Muc2* and *ZO-1*gene in colon tissues of young and aged mice.
4. Plasma endotoxin level in young and aged mice.

Data were represented as mean ± SD. **p* < 0.05, ***p* < 0.01, and ****p* < 0.001; ns, not significant.

**Table S1**

| *Akkermansia muciniphila*-F | CAGCACGTGAAGGTGGGGAC |
| --- | --- |
| *Akkermansia muciniphila*-R | CCTTGCGGTTGGCTTCAGAT |
| universal Eubacteria 16S-F | CGGCAACGAGCGCAACCC |
| universal Eubacteria 16S-R | CCATTGTAGCACGTGTGTAGCC |
| m-Muc2-F | AGGGCTCGGAACTCCAGAAA |
| m-Muc2-R | CCAGGGAATCGGTAGACATCG |
| m-Ocln-F | TTGAAAGTCCACCTCCTTACAGA |
| m-Ocln-R | CCGGATAAAAAGAGTACGCTGG |
| m-Il6-F | CCAAGAGGTGAGTGCTTCCC |
| m-Il6-R | CTGTTGTTCAGACTCTCTCCCT |
| m-IL-1β-F | GAAATGCCACCTTTTGACAGTG |
| m-IL-1β-R | TGGATGCTCTCATCAGGACAG |
| m-Tnf-α-F | CTGAACTTCGGGGTGATCGG |
| m-Tnf-α-R | GGCTTGTCACTCGAATTTTGAGA |
| m-Gria1-F | ACTGGAAGAGGCCAAAGTACAC |
| m-Gria1-R | CTGGGTTAGCCAGACAGTCC |
| m-Gria2-F | TTCTCCTGTTTTATGGGGACTGA |
| m-Gria2-R | CTACCCGAAATGCACTGTATTCT |
| m-CamKIIα-F | ACCTGCACCCGATTCACAG |
| m-CamKIIα-R | TGGCAGCATACTCCTGACCA |
| m-CamKIIβ-F | TCACCGACGAGTACCAGCTA |
| m-CamKIIβ-R | GGCAGATCCGAGCTTCTCTC |
| m-*Bdnf*-F | TCATACTTCGGTTGCATGAAGG |
| m-*Bdnf*-F | AGACCTCTCGAACCTGCCC |
| m-Ngf-F | AGACTCCACTCACCCCGTG |
| m-Ngf-R | GGCTGTGGTCTTATCTCCAAC |
| m-Nos1-F | CTGGTGAAGGAACGGGTCAG |
| m-Nos1-R | CCGATCATTGACGGCGAGAAT |
| m-Nos2-F | GTTCTCAGCCCAACAATACAAGA |
| m-Nos2-R | GTGGACGGGTCGATGTCAC |
| m-Nos3-F | GGCTGGGTTTAGGGCTGTG |
| m-Nos3-R | CTGAGGGTGTCGTAGGTGATG |
| m-Sod1-F | AACCAGTTGTGTTGTCAGGAC |
| m-Sod1-R | CCACCATGTTTCTTAGAGTGAGG |
| m-Cat-F | AGCGACCAGATGAAGCAGTG |
| m-Cat-R | TCCGCTCTCTGTCAAAGTGTG |
| m-*H0-1*-F | AAGCCGAGAATGCTGAGTTCA |
| m-*H0-1*-F | GCCGTGTAGATATGGTACAAGGA |
| m-*Gpx*-F | CCTTTTAAGCAGTATGCAGGCA |
| m-*Gpx*-F | CAAGCCAAATGGCCCAAGTT |
| m-Lgr5-F | GACAATGCTCTCACAGAC |
| m-Lgr5-R | GGAGTGGATTCTATTATTATGG |
| m-Actin-F | GAGACCTTCAACACCCCAGC |
| m-Actin-R | GGAGAGCATAGCCCTCGTAGAT |
